# SPACE: A novel digital tool for assessing hippocampal structural integrity in older adults

**DOI:** 10.1038/s41598-026-39628-8

**Published:** 2026-02-12

**Authors:** Karolina Minta, Giorgio Colombo, Mervin Tee, Marcus Low, Jascha Grübel, Jan Wiener, Christopher P. Chen, Saima Hilal, Victor R. Schinazi

**Affiliations:** 1 Future Health Technologies, Singapore-ETH Centre, Campus for Research Excellence and Technological Enterprise (CREATE), Singapore, Singapore; 2 Department of Pharmacology, Yong Loo Lin School of Medicine, National University of Singapore, Singapore, 117600 Singapore; 3 Chair of Cognitive Science, ETH Zurich, Zürich, 8092 Switzerland; 4 Saw Swee Hock School of Public Health, National University of Singapore, National University Health System, Singapore, Singapore; 5 Center for Sustainable Future Mobility, ETH Zurich, Zürich, 8092 Switzerland; 6 Laboratory of Geo-information Science and Remote Sensing, Wageningen University, Wageningen, 6708 PB The Netherlands; 7 Department of Psychology, Ageing & Dementia Centre, Bournemouth University, Poole, UK; 8 Memory Aging and Cognition Centre, National University Health System, Singapore, 117600 Singapore; 9 Department of Psychology, Bond University, Gold Coast, QLD Australia

**Keywords:** Cognitive assessment, Cognitive map, Hippocampus, MRI, Spatial navigation, Volumetry, Cognitive ageing, Human behaviour

## Abstract

Hippocampal atrophy is a hallmark of Alzheimer’s disease and is linked to deficits in navigation. We investigated whether performance in a novel digital assessment, the Spatial Performance Assessment for Cognitive Evaluation (SPACE), is associated with hippocampal volume beyond traditional neuropsychological tests in older adults. Forty older adults (M_age_ = 67, SD = 6) underwent structural MRI and completed the spatial and navigation tasks in SPACE along with a battery of neuropsychological tests typically used to detect cognitive impairment. Regression analyses revealed that poorer performance in the path integration and mapping tasks was associated with smaller hippocampal volume after accounting for age, education, and neuropsychological test performance. Notably, individuals who accurately completed the path integration task and successfully learned the spatial configuration of landmarks required for subsequent reconstruction in the mapping task exhibited larger hippocampal volumes. Together, these findings suggest that SPACE may capture aspects of spatial cognition closely linked to hippocampal structural integrity and may complement existing cognitive assessments by providing increased sensitivity to hippocampal variation in non-clinical older adults.

**Supplementary Information:**

The online version contains supplementary material available at 10.1038/s41598-026-39628-8.

## Introduction

Ageing is associated with structural changes in the medial temporal lobe^1–5^, with atrophy of structures in this region being a hallmark of pathological cognitive decline^6^. The medial temporal lobe also plays an instrumental role in supporting navigation^7–10^. Specifically, place cells in the hippocampus^11,12^ and grid cells in the entorhinal cortex^13,14^ are essential for coding locations and tracking changes in position and orientation during navigation, respectively. Researchers have also identified homologues of these cells supporting human navigation^15,16^, and neuroimaging evidence suggests that the hippocampus is essential for place learning and goal-directed navigation by encoding spatial information into flexible representations known as cognitive maps^17,18^. Here, studies have found that the hippocampus is active during recall of complex routes around the city^19^ and is associated with navigation accuracy in complex Virtual Environments (VE)^20^. Similarly, studies have shown that hippocampal activity is correlated with acquired knowledge during navigational learning^21^ and modulated by the distance to goal locations^22–24^. The hippocampus is also implicated in Path Integration (PI)^25–27^, especially in situations where long-term memory requirements are high^28^ and paths are complex^29^. In these contexts, the hippocampus supports both the updating of self-motion and the integration of spatial representations, although these functions can draw on a broader, distributed network, including the entorhinal cortex^30,31^, the caudate^32,33^, the prefrontal cortex^34,35^, and the human motion complex^27^.

Results from structural brain imaging studies largely corroborate these findings. Studies have shown that the hippocampus of taxi drivers with extensive navigation experience is larger than that of controls^36^ and bus drivers^37^, the latter of whom typically follow a constrained set of routes as part of their job. Interestingly, hippocampal volume also correlates with the time spent learning to be a taxi driver^36^ and with successfully completing a taxi training program^38^. Other research suggests that structural changes in the hippocampus may not be specific to expert taxi drivers but to the ability to build flexible representations during navigation^39,40^. For example, hippocampal volume has been shown to correlate with pointing accuracy in large-scale real-world navigation tasks^39^ as well as with the ability to learn and flexibly navigate routes in VEs^40^.

Research with healthy older adults has also shown that hippocampal volume correlates with spatial memory^41^ and navigation performance^42,43^ and may be specific to spatial^44–47^ but not response strategies^47^. For example, Driscoll and colleagues^44^ found that larger hippocampal volumes were associated with better performance in a virtual Morris Water Maze task. Similarly, Korthauer and colleagues^45^ reported positive associations between performance in the virtual Morris Water Maze and hippocampal volume after age correction. Using a radial maze, Konishi and Bohbot also found that, although performance did not differ, hippocampal volume correlated with the use of spatial strategies^46^. These results were further supported by Sodums and Bohbot^47^, who found that spatial strategies were positively associated with hippocampal volume, while response strategies were positively associated with the caudate. The relationship between hippocampal atrophy and navigation ability is more pronounced in individuals with Mild Cognitive Impairment (MCI) and Alzheimer’s Disease (AD)^31,48–50^. For example, healthy controls outperformed AD patients on spatial recall, but the relationship between task performance and hippocampal volume was significant only in AD patients^48^. Similarly, smaller hippocampal volume has been linked to poorer navigation performance in both real and virtual spaces among MCI and AD patients, but not in cognitively healthy individuals^49^.

Despite these findings, the relationship between hippocampal volume and navigation ability remains controversial^51–53^ and may depend on gender^54^. Two large-sample studies with non-expert navigators did not find a significant relationship between hippocampal volume and various navigation tasks after active^53^ or passive^52^ learning in a VE. However, a recent study^55^ that reanalysed data from Weisberg and colleagues^53^ found that the relationship between hippocampal volume and spatial learning is significantly stronger in individuals with high spatial ability and is moderated by self-reported sense of direction and cognitive map formation. The inconsistency in results across studies may also be due to disagreement among researchers about which tests to use and their relative sensitivities for capturing hippocampal atrophy^54^.

To date, there is a lack of non-invasive, cost-effective screening measures that can accurately reflect structural changes in the brain. There is some evidence that neuropsychological tests of cognitive functioning may be used to relate cognitive impairment to morphological changes. The Montreal Cognitive Assessment (MoCA) is a widely used tool for discriminating between healthy individuals and patients with MCI^56^. Despite the widespread use of the MoCA, comparatively few studies have examined its neuroanatomical correlates^57–66^, and an even smaller subset has focused specifically on hippocampal structure or on associations with individual MoCA subdomains^60–66^. Some studies have reported that lower total MoCA scores are associated with hippocampal atrophy, but these findings have primarily been observed in clinical or at-risk populations, including individuals with subjective cognitive impairment^61^ or patients with established cognitive impairment^62–65^. In non-clinical populations, evidence is more heterogeneous. Paul and colleagues reported an association between hippocampal volume and the MoCA naming subdomain, but not with the total MoCA score^60^. Similarly, Gupta and colleagues observed associations between hippocampal volume and both the MoCA total score and several subdomains, with the strongest relationship observed for visuospatial function, followed by attention, orientation, and verbal memory^66^. These latter findings suggest that, in healthy adults, both the total MoCA score and its subdomains may be informative when relating cognitive performance to hippocampal structure.

Since only some neuropsychological tests and a small section of the MoCA assess visuospatial abilities, administering a more comprehensive battery of spatial navigation tasks as a cognitive assessment may improve accuracy in detecting associated structural changes in the hippocampus. In this study, we deployed a novel digital tool, the Spatial Performance Assessment for Cognitive Evaluation (SPACE), which assesses various aspects of spatial navigation within a single VE^67–70^. SPACE begins with a PI task, in which participants encode the spatial layout of landmarks through self-motion, followed by a series of tasks (i.e., pointing, mapping, and perspective taking) that require recalling, transforming, and reconstructing this information. This structure allows us to dissociate the accuracy of spatial encoding in PI from the fidelity with which that information is later reconstructed into allocentric representations in the subsequent tasks. Using structural MRI, we examined whether performance in SPACE is more strongly associated with hippocampal volume than the MoCA and other standard neuropsychological tests. We hypothesised that hippocampal volume would be most strongly associated with the joint performance of PI and subsequent spatial reconstruction. Specifically, individuals who showed both low PI error and high accuracy in the downstream navigation tasks (i.e., pointing and mapping) were expected to exhibit larger hippocampal volumes, whereas poorer joint performance was expected to be associated with reduced hippocampal volume. If successful, SPACE may be used as a complementary, non-invasive, cost-effective screening tool to assess cognitive functioning and its neural correlates in healthy older adults.

## Results

**Fig. 1:**
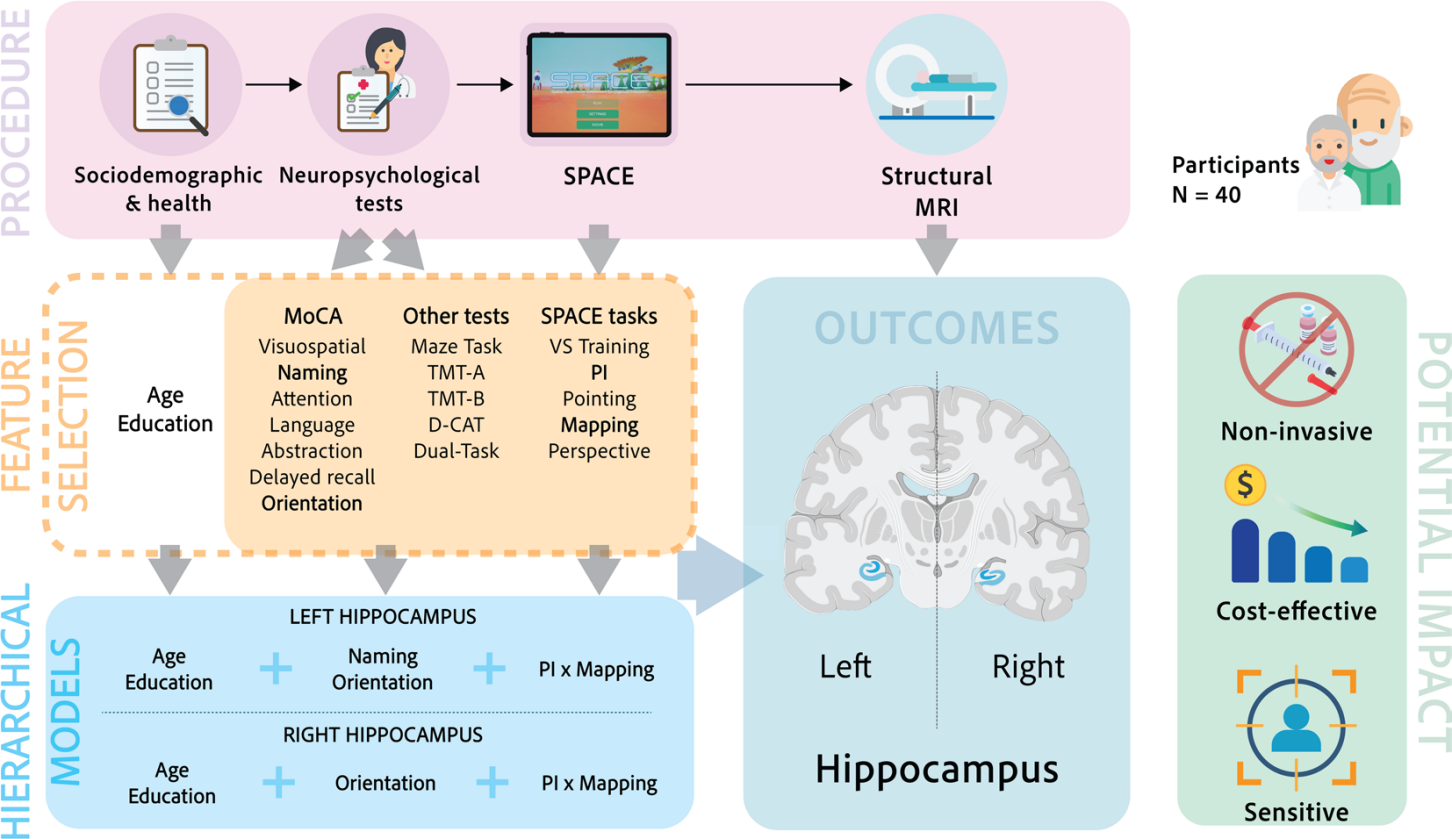
Study design and analytical framework. A graphical representation of the study design and regression models examining the association between hippocampal volume, performance on standard neuropsychological tests, and SPACE tasks. Forty older adults completed a sociodemographic and health questionnaire, a neuropsychological battery, and SPACE, followed by structural MRI. Feature selection analyses were used to identify predictors of left and right hippocampal volume, controlling for age and education. Hierarchical regression models were then specified with age and education entered in the first block, followed by feature-selected MoCA subdomains and a composite SPACE measure capturing joint path-integration and mapping performance (PI × Mapping) in subsequent blocks.

Forty older male participants completed the sociodemographic and health questionnaire, the neuropsychological test battery, and the SPACE tasks before undergoing an MRI scan. Table 1 presents the demographic information, neuroimaging characteristics, and scores for the neuropsychological assessments and the tasks in SPACE. Figure 1 presents the procedure, and additional details are provided in the Methods section.

**Table 1:** Demographic data, neuroimaging characteristics, neuropsychological test and SPACE scores.

Variable	Value (*N* = 40)	
	Mean (SD)/%	Median (Min - Max)
Age *(years)*	67 (6)	67 (55–79)
Education *(%)*		
Secondary or below	40%	
Postsecondary	60%	
Brain volume		
Total Gray matter *(mm*^*3*^*)*	461,599 (44,417)	462,694 (379,786 − 542,369)
Hippocampal left *(mm*^*3*^*)*	2,603 (306)	2,569 (2,097 − 3,191)
Hippocampal right *(mm*^*3*^*)*	2,653 (337)	2,629 (1,879–3,246)
Entorhinal cortex left *(mm*^*3*^*)*	1,367 (427)	1,327 (566–2,555)
Entorhinal cortex right *(mm*^*3*^*)*	1,452 (356)	1,498 (455–2,079)
Neuropsychological assessments		
MoCA	27 (3)	28 (20–30)
Maze Task *(seconds)*	29 (11)	26.5 (15–60)
D-CAT *(counts)*	29 (7)	29 (11–40)
TMT-A *(seconds)*	42 (24)	37.5 (18–144)
TMT-B *(seconds)*	121 (95)	91.5 (39–540)
Dual-Task *(%)*	99 (14)	99 (67.64–145.23)
SPACE		
Visuospatial training *(seconds)*	259.63 (40.10)	247.94 (207.70–419.05)
PI distance error *(meters)*	213.38 (117.44)	189.91 (116.57–806.86)
Pointing error *(degrees)*	78.80 (15.14)	77.27 (49.61–112.83)
Mapping performance *(r*^*2*^*)*	0.51 (0.31)	0.52 (0.03–1.00)
Perspective taking error *(degrees)*	38.80 (25.95)	38.15 (5.75–117.30)

Abbreviations: MoCA: Montreal Cognitive Assessment; D-CAT: Digit Cancellation Test; TMT: Trail Making Test; SPACE: Spatial Performance Assessment for Cognitive Evaluation; PI: Path Integration. The secondary or below education level included participants with no formal education (*n* = 1), primary (*n* = 6) and secondary (*n* = 9) education. The postsecondary education level included participants with junior college (*n* = 1), polytechnic (*n* = 8), and university degrees (*n* = 15).

### Feature selection for the MoCA, neuropsychological battery and SPACE

To assess which MoCA subdomains were most strongly associated with left and right hippocampal volumes (Table 2), we fitted multiple regression models that included the scores for each MoCA subdomain, along with age and education. The overall models were statistically significant for both the right hippocampus, *F*(9, 30) = 4.38, *p* = 0.001, *R*² = 0.57 (adjusted *R*² = 0.44), and the left hippocampus, *F*(9, 30) = 2.93, *p* = 0.013, *R*² = 0.47 (adjusted *R*² = 0.31), indicating moderate to substantial explained variance in hippocampal volume. Within these models, only age (*β* = −0.41, *p* = 0.007) and the MoCA orientation subdomain (*β* = 0.27, *p* = 0.039) explained unique variance in right hippocampal volume, whereas the MoCA naming (*β* = −0.40, *p* = 0.014) and orientation subdomains (*β* = 0.31, *p* = 0.034) explained unique variance in left hippocampal volume. All other MoCA subdomains did not show any significant associations (all *p* ≥ 0.099).

**Table 2:** Featureselection analysis of MoCA subdomains associated with hippocampal volume.

Predictor	Left hippocampus				Right hippocampus			
	Est.	SE	t	*p*	Est.	SE	t	*p*
	R^2^ = 0.47/R^2^_adj_ = 0.31* (f^2^= 0.89)				R^2^ = 0.57/R^2^_adj_ = 0.44** (f^2^ = 1.33)			
*Intercept*	*2996.48*	*353.23*	*2.214*	*0.035*	*1133.03*	*1347.63*	*0.841*	*0.407*
Age	−13.38	7.96	−1.681	0.103	**−23.08**	**7.92**	**−2.912**	**0.007**
Education	108.38	124.67	0.869	0.392	163.73	124.16	1.319	0.197
Visuospatial	103.84	72.32	1.436	0.161	122.44	72.02	1.7	0.099
Naming	**−772.13**	**295.25**	**−2.615**	**0.014**	136.22	294.03	0.463	0.647
Attention	47.94	53.65	0.894	0.379	−59.07	53.43	−1.106	0.278
Language	−13.57	97.49	−0.139	0.890	140.1	97.09	1.443	0.159
Abstraction	−105.17	83.65	−1.257	0.218	−133.68	83.31	−1.605	0.119
Delayed recall	33.66	37.12	0.907	0.372	37.68	36.97	1.019	0.316
Orientation	**354.59**	**159.14**	**2.228**	**0.034**	**341.39**	**158.48**	**2.154**	**0.039**

Abbreviations: Est.: Estimate; SE: Standard Error. The variables with a significant (*p* < 0.05) contribution to the model are marked in bold, and effect sizes are provided as Cohen’s f^2^. For the model fit measures, the number of stars designates the significance level: *** < 0.001, ** < 0.01, * < 0.05.

We applied the same feature selection approach to the battery of neuropsychological tests by simultaneously entering all test measures into multiple regression models, along with age and education (Table 3). The overall model was statistically significant for the right hippocampus, *F*(7, 32) = 3.13, *p* = 0.012, explaining a moderate proportion of variance (*R*² = 0.41, adjusted *R*² = 0.28). In contrast, the corresponding model for the left hippocampus was not statistically significant, *F*(7, 32) = 1.63, *p* = 0.162. None of the individual neuropsychological measures explained unique variance in hippocampal volume after accounting for age and education (all *p* ≥ 0.091 for the right hippocampus; all *p* ≥ 0.140 for the left hippocampus). This pattern suggests substantial shared variance across the neuropsychological measures, rather than distinct domain-specific associations with hippocampal volume when all tasks are considered simultaneously.

**Table 3:** Featureselection analysis of standard neuropsychological test measures associated with hippocampal volume.

Predictor	Left hippocampus				Right hippocampus			
	Est.	SE	t	*p*	Est.	SE	t	*p*
	R^2^ = 0.26/R^2^_adj_ = 0.10				R^2^ = 0.41/R^2^_adj_ = 0.28* (f^2^ = 0.68)			
Intercept	*3421.83*	*693.85*	*4.932*	*< 0.001*	*3526.13*	*687.77*	*5.127*	*< 0.001*
Age	−12.74	9.32	−1.367	0.181	−17.45	9.24	−1.889	0.068
Education	134.85	128.69	1.048	0.303	222.54	127.56	1.745	0.091
Maze Task	−7.01	5.57	−1.257	0.218	−4.29	5.53	−0.760	0.453
TMT-A	5.04	3.33	1.513	0.140	1.52	3.31	0.459	0.649
TMT-B	−0.55	0.74	−0.733	0.469	0.08	0.74	0.115	0.910
D-CAT	2.34	8.81	0.265	0.793	9.5	8.73	1.093	0.283
Dual-Task	−0.65	3.82	−0.170	0.866	−0.71	3.78	−0.188	0.852

Abbreviations: Est.: Estimate; SE: Standard Error; TMT: Trail Making Test; D-CAT: Digit Cancellation Test. The variables with a significant (*p* < 0.05) contribution to the model are marked in bold, and effect sizes are provided as Cohen’s f^2^. For the model fit measures, the number of stars designates the significance level: *** < 0.001, ** < 0.01, * < 0.05.

We next applied the same feature selection approach to the tasks in SPACE by simultaneously entering visuospatial training time, PI distance error, pointing error, mapping performance, and perspective taking error into multiple regression models, along with age and education (Table 4). The overall models were statistically significant for both hemispheres. For the right hippocampus, the model explained a substantial proportion of variance, *F*(7, 32) = 5.39, *p* < 0.001, *R*² = 0.54 (adjusted *R*² = 0.44). Similarly, the model for the left hippocampus explained a substantial proportion of variance, *F*(7, 32) = 5.10, *p* < 0.001, *R*² = 0.53 (adjusted *R*² = 0.42). Within these models, education was positively associated with hippocampal volume for both the right (*β* = 0.78, *p* = 0.018) and left hippocampus (*β* = 0.86, *p* = 0.011). In addition, poorer PI performance was associated with smaller hippocampal volumes on both the right (*β* = −0.32, *p* = 0.021) and left (*β* = −0.33, *p* = 0.020) hemispheres, while poorer mapping performance was associated with larger hippocampal volume for both the right (*β* = −0.35, *p* = 0.008) and left hemispheres (*β* = −0.54, *p* < 0.001). Visuospatial training time, pointing error, and perspective taking error did not explain unique variance in hippocampal volume after accounting for age and education (all *p* ≥ 0.13).

**Table 4:** Featureselection analysis of SPACE associated with hippocampal volume.

Predictor	Left hippocampus				Right hippocampus			
	Est.	SE	t	p	Est.	SE	t	p
Main effects model	R^2^ = 0.53/R^2^_adj_ =0.42*** (f^2^ = 1.11)				R^2^ = 0.54/R^2^_adj_ =0.44*** (f^2^ = 1.18)			
Intercept	3286.33	558.31	5.886	< 0.001	4071.84	607.62	6.701	< 0.001
Age	-12.02	7.76	-1.55	0.131	-15.21	8.44	-1.802	0.081
Education	**263.07**	**97.4**	**2.7**	**0.011**	**263.12**	**106**	**2.482**	**0.018**
VS Training	1.65	1.06	1.556	0.130	-0.01	1.15	-0.009	0.993
PI distance	**-0.86**	**0.35**	**-2.444**	**0.020**	**-0.93**	**0.38**	**-2.436**	**0.021**
Pointing	-0.89	2.88	-0.309	0.759	-2.12	3.14	-0.676	0.504
Mapping	**-532.58**	**125.2**	**-4.253**	**< 0.001**	**-384.78**	**136.29**	**-2.823**	**0.008**
Perspective	1.38	1.61	0.859	0.397	-0.02	1.75	-0.013	0.990
Composite model 1	R^2^ = 0.21/R^2^_adj_ =0.14				R^2^ = 0.40/R^2^_adj_ =0.35*** (f^2^ = 0.66)			
Intercept	3408	603.5	5.648	< 0.001	3,762	580	6.49	< 0.001
Age	-12.48	8.742	-1.428	0.162	-16.89	8.402	-2.01	0.052
Education	152.9	103.3	1.48	0.148	**247.1**	**99.27**	**2.49**	**0.018**
PI × Pointing	-0.004	0.006	-0.63	0.532	-0.008	0.006	-1.32	0.196
Composite model 2	R^2^ = 0.47/R^2^_adj_ =0.42*** (f^2^ = 0.87)				R^2^ = 0.51/R^2^_adj_ =0.47*** (f^2^ = 1.04)			
Intercept	3523	495.5	7.11	< 0.001	3862	524.4	7.36	< 0.001
Age	-11.7	7.034	-1.66	0.105	**-17.62**	**7.445**	**-2.37**	**0.023**
Education	**175.8**	**84.76**	**2.07**	**0.045**	**268.8**	**89.71**	**3**	**0.005**
PI × Mapping	**-2.426**	**0.57**	**-4.26**	**< 0.001**	**-1.93**	**0.603**	**-3.2**	**0.003**

Est.: Estimate; SE: Standard Error; VS: Visuospatial; PI: Path Integration. The variables with a significant (p < 0.05) contribution to the model are marked in bold, and effect sizes are provided as Cohen’s f^2^. For the model fit measures, the number of stars designates the significance level: *** < 0.001, ** < 0.01, * < 0.05.

Finally, we examined whether combining interdependent SPACE tasks captured additional variance in hippocampal volume beyond their individual associations by testing synergy models between PI distance error and either pointing error or mapping performance. Models including the interaction term between PI distance error and pointing error were statistically significant for both hemispheres. For the right hippocampus, education emerged as the only significant contributor (*β* = 0.60, *p* = 0.018), with the overall model explaining 40% of the variance, *F*(3, 36) = 7.96, *p* < 0.001 (adjusted *R*² = 0.35). For the left hippocampus, the overall model was statistically significant, *F*(3, 36) = 3.11, *p* = 0.038, explaining a small proportion of variance (*R*² = 0.21, adjusted *R*² = 0.14), but no individual predictors reached statistical significance (all *p* > 0.148).

**Fig. 2:**
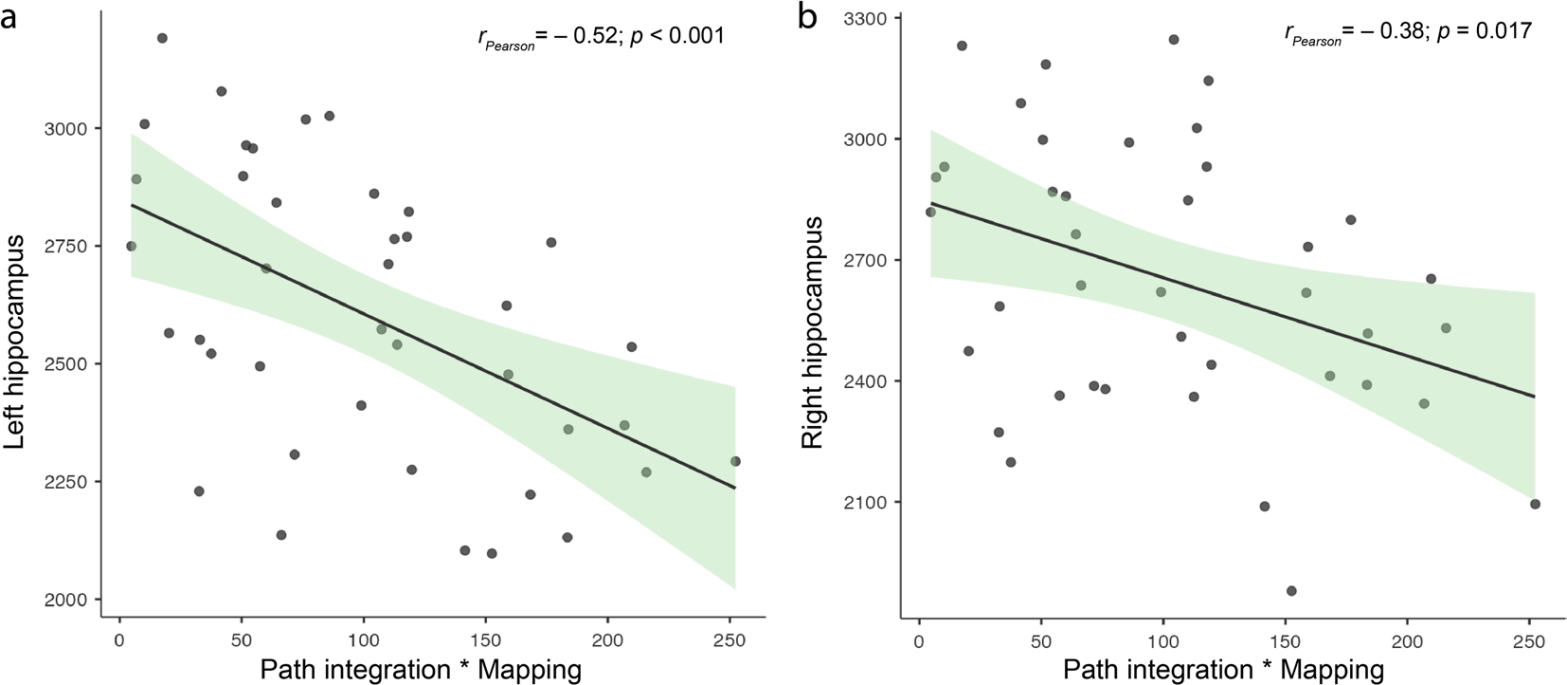
Interaction effects. Scatter plots show the relationship between hippocampal volume and the interaction between PI distance error and mapping performance for the left (a) and right (b) hippocampus in a sample of 40 participants.

Models including the interaction term between PI distance error and mapping performance were statistically significant for both hemispheres. For the right hippocampus, age (*β* = −0.34, *p* = 0.023), education (*β* = 0.74, *p* = 0.005), and the interaction term (*β* = 0.21, *p* = 0.003) explained 51% of the variance, *F*(3, 36) = 12.47, *p* < 0.001 (adjusted *R*² = 0.47). For the left hippocampus, education (*β* = 0.50, *p* = 0.045) and the interaction term (*β* = 0.19, *p* < 0.001) jointly explained 47% of the variance in hippocampal volume, *F*(3, 36) = 10.47, *p* < 0.001 (adjusted *R*² = 0.42). Figure 2 illustrates that reduced hippocampal volumes were observed when imprecision in PI co-occurred with poorer mapping performance (see Supplementary Information 5).

### Hierarchical regression analyses

To evaluate whether joint performance on PI and mapping explains additional variance in hippocampal volume beyond demographics and standard cognitive measures, we conducted separate hierarchical regression models for the left and right hippocampus (Table 5). Here, predictors were entered in steps informed by the preceding feature selection analyses.

For the right hippocampus, age and education were entered in the first step and explained 37% of the variance in hippocampal volume, *F*(2, 37) = 10.85, *p* < 0.001 (adjusted *R*² = 0.34). In the second step, the addition of the MoCA orientation subdomain accounted for an additional 8% of explained variance (*p* = 0.025), resulting in a model explaining 45% of the variance, *F*(3, 36) = 9.91, *p* < 0.001 (adjusted *R*² = 0.41). In the final step, the inclusion of the PI × mapping interaction term explained a further 7% of the variance (*p* = 0.028), yielding a final model that explained 52% of the variance, *F*(4, 35) = 9.63, *p* < 0.001 (adjusted *R*² = 0.47). Within this model, age (*β* = −0.34, *p* = 0.021), education (β = 0.76, *p* = 0.005), and the PI × mapping interaction term (*β* = 0.23, *p* = 0.028) were all independently associated with hippocampal volume.

**Table 5:** Hierarchical regression models of hippocampal volume with feature-selected MoCA variables and the SPACE interaction.

Predictor	Left hippocampus				Right hippocampus			
	Est.	SE	t	P	Est.	SE	t	p
Step 1	R^2^=0.20/R^2^_adj_=0.15*(f^2^= 0.25)				R^2^= 0.37/R^2^_adj_= 0.34***(f^2^= 0.59)			
Intercept	3415.46	598.44	5.71	<.001	3776.494	585.628	6.449	<0.001
Age	-13.6	8.49	-1.6	0.118	**-19.132**	**8.308**	**-2.303**	**0.027**
Education	155.62	102.35	1.52	0.137	**252.665**	**100.156**	**2.523**	**0.016**
Step 2	R^2^=0.40/R^2^_adj_=0.33**(f^2^=0.67)				R^2^=0.45/R^2^_adj_=0.41**(f^2^=0.82)			
Intercept	2860.014	1305.151	2.191	0.035	1634.101	1072.591	1.524	0.136
Age	-13.885	7.574	-1.833	0.075	**-19.409**	**7.852**	**-2.472**	0.018
Education	**197.318**	**92.737**	**2.128**	**0.040**	**258.704**	**94.677**	**2.732**	**0.01** **0**
Naming	**-567.021**	**259.115**	**-2.188**	**0.035**	-	-	-	-
Orientation	**377.459**	**150.738**	**2.504**	**0.017**	**364.088**	**156.147**	**2.332**	**0.025**
Step 3	R^2^=0.55/R^2^_adj_=0.48*** (f^2^= 1.22)				R^2^=0.52 /R^2^_adj_=0.47 *** (f^2^= 1.08)			
Intercept	4329.987	1223.72	3.538	0.001	2822.502	1139.12	2.478	0.018
Age	-12.054	6.664	-1.809	0.079	**-18.027**	**7.45**	**-2.42**	**0.021**
Education	**208.496**	**81.388**	**2.562**	**0.015**	**268.743**	**89.641**	**2.998**	**0.005**
Naming	-**532.756**	**227.443**	**-2.342**	**0.025**	-	-	-	-
Orientation	126.362	151.483	0.834	0.410	174.063	169.34	1.028	0.311
PI × Mapping	**-2.1**	**0.619**	**-3.394**	**0.002**	**-1.584**	**0.691**	**-2.292**	**0.028**
Model comparison								
	Left hippocampus				Right hippocampus			
Models	∆R^2^	F	df1/df2	p	∆R^2^	F	df1/df2	p
1-2	**0.199**	**5.751**	2/35	**0.007**	**0.083**	**5.437**	1/36	**0.025**
2-3	**0.153**	**11.516**	1/34	**0.002**	**0.071**	**5.255**	1/36	**0.028**

For the left hippocampus, age and education were entered in the first step and explained 20% of the variance in hippocampal volume, *F*(2, 37) = 4.54, *p* = 0.017 (adjusted *R*² = 0.15). In the second step, the MoCA subdomains identified in the feature selection analysis (i.e., naming and orientation) accounted for an additional 20% of explained variance (*p* = 0.007), improving overall model fit, *F*(4, 35) = 5.73, *p* = 0.001 (adjusted *R*² = 0.33). In the final step, inclusion of the PI × mapping interaction term explained a further 15% of the variance (*p* = 0.002), yielding a final model that explained 55% of the variance, *F*(5, 34) = 8.26, *p* < 0.001 (adjusted *R*² = 0.49). Within this model, education (*β* = 0.64, *p* = 0.015), MoCA naming (*β* = −0.29, *p* = 0.025), and the PI × mapping interaction term (*β* = 0.20, *p* = 0.002) were independently associated with hippocampal volume.

As a robustness check, we additionally conducted hierarchical regression models for the left and right hippocampus in which the total MoCA score was entered in place of the feature-selected MoCA subdomains. The pattern of results remained unchanged, with the PI × mapping interaction explaining significant additional variance in hippocampal volume (see Supplementary Table 5).

### Entorhinal cortex volume and spatial navigation performance

Given the complementary roles that the hippocampus and the entorhinal cortex play in navigation, we applied the same feature selection strategy to examine whether MoCA measures, standard neuropsychological tests, or SPACE performance were associated with entorhinal cortex volume, controlling for age and education (see Supplementary Information 1). Models including individual the MoCA subdomains were not statistically significant for either the right, *F*(9, 30) = 1.79, *p* = 0.111, or the left, *F*(9, 30) = 2.11, *p* = 0.061, entorhinal cortex. When examining the total MoCA score, regression models were statistically significant for the right *F*(3, 36) = 3.37, *p* = 0.029, *R*² = 0.22 (adjusted *R*² = 0.15) and left *F*(3, 36) = 3.05, *p* = 0.041, *R*² = 0.20 (adjusted *R*² = 0.14) entorhinal cortex. However, within these models, neither age, education, nor total MoCA score explained unique variance (all *p* ≥ 0.053). Featureselection analyses of the standard neuropsychological battery did not yield significant models for the left entorhinal cortex, *F*(7, 32) = 1.35, *p* = 0.261. For the right entorhinal cortex, the overall model reached statistical significance, *F*(7, 32) = 3.31, *p* = 0.009, *R*² = 0.42 (adjusted *R*² = 0.29). However, only TMT-A performance showed a significant association (*β* = 0.56, *p* = 0.020), and none of the other neuropsychological measures explained unique variance after accounting for age and education.

Featureselection analyses of SPACE task performance revealed no statistically significant associations between individual navigation measures and entorhinal cortex volume for either the right *F*(7, 32) = 1.94, *p* = 0.095, or the left *F*(7, 32) = 1.83, *p* = 0.116, entorhinal cortex. We also examined whether joint efficiency models, captured by the interaction term between path-integration distance error and either pointing error or mapping performance, were associated with entorhinal cortex volume. Models including the PI × pointing interaction term were statistically significant at the model level for both the right, *F*(3, 36) = 3.20, *p* = 0.035, *R*² = 0.21 (adjusted *R*² = 0.15), and the left, *F*(3, 36) = 3.31, *p* = 0.031, *R*² = 0.22 (adjusted *R*² = 0.15), entorhinal cortex. However, in neither model did demographics or the interaction term explain unique variance (all *p* ≥ 0.07). Similarly, models including the PI × mapping interaction term were statistically significant at the model level for both the right, *F*(3, 36) = 3.17, *p* = 0.036, *R*² = 0.21 (adjusted *R*² = 0.14), and the left, *F*(3, 36) = 3.59, *p* = 0.02, *R*² = 0.23 (adjusted *R*² = 0.17), entorhinal cortex. However, only age showed a significant association with right entorhinal cortex volume (*β* = −0.35, *p* = 0.049). Because none of the SPACE measures survived feature selection for the entorhinal cortex, hierarchical regression analyses analogous to those conducted for the hippocampus were not warranted.

## Discussion

We investigated whether performance on standard neuropsychological tests and a novel digital assessment of navigation ability (SPACE) was associated with hippocampus volume in healthy older adults. We found that only a limited subset of MoCA subdomains showed independent associations with hippocampal volume after feature selection, and the broader neuropsychological test battery did not provide comparable explanatory power. In contrast, performance on the PI and mapping tasks in SPACE was associated with hippocampal volume after controlling for age and education. Specifically, participants who accurately completed the PI task and successfully learned the spatial configuration of landmarks required for subsequent reconstruction in the mapping task exhibited larger hippocampal volumes. Hierarchical regression analyses further showed that the joint efficiency of path integration and mapping performance explained additional variance in hippocampal volume beyond age, education, and feature-selected MoCA subdomains, with the full models accounting for 52% and 55% of the variance in right and left hippocampal volume, respectively.

As expected, older age was associated with reduced hippocampal and entorhinal cortex volumes. These findings are consistent with well-established research documenting age-related atrophy in medial temporal lobe structures^71–76^. Brain volume reductions have been observed in individuals as young as 30, with the rate of atrophy accelerating with age^72–74^. In healthy ageing, the average annual volume reduction has been estimated at approximately 0.9% for the hippocampus and 1.3% for the entorhinal cortex^72^. Critically, this rate is reported to be almost 6% in the hippocampus and above 7% in the entorhinal cortex in AD patients^72^. Accordingly, medial temporal lobe atrophy is a defining feature of AD and is often used to distinguish patients with MCI and AD from healthy ageing^77^. Here, research by Henneman and colleagues showed that the hippocampal atrophy rate is more suitable than whole-brain volume for distinguishing between MCI and controls, and that estimating hippocampal volume may be useful for measuring the progression of cognitive impairment^76^. We also found that higher educational attainment was associated with larger hippocampal and entorhinal cortex volumes. Hippocampal volume is known to vary with educational attainment across the lifespan^78^, and this relationship may be particularly strong in individuals with AD^79,80^. In contrast, the relationship between education and the entorhinal cortex is unclear. Although most studies account for educational level in regression models, some studies have not found a link between entorhinal cortex volume and education^81,82^.

Among the SPACE tasks, the PI and mapping tasks were the only significant predictors of left and right hippocampal volume. More importantly, hippocampal volume was most strongly associated with the joint performance of these two tasks, operationalised as their multiplicative combination. This is understandable, as successful map construction depended on the accurate acquisition of spatial information during the PI task. Participants who showed both low PI distance error and high mapping accuracy exhibited the largest hippocampal volumes, whereas poorer combined performance was associated with reduced hippocampal volume. Namely, mapping performance contributed positively to hippocampal volume only when preceded by accurate PI, indicating that faithful landmark encoding was necessary for later reconstruction to reflect hippocampal integrity. By capturing the co-occurrence of efficient encoding and reconstruction, this joint metric may help clarify previously inconsistent findings on the relationship between navigation task performance and hippocampal volume^39,52,53,55^, and suggest that hippocampal navigation relationships are strongest when multiple, complementary aspects of spatial ability are considered together rather than in isolation.

In the model without the joint term, PI error and mapping performance were both associated with hippocampal volume, albeit in opposite directions. Previous research has shown that the hippocampus plays an important role in successful PI^25,83^. For example, hippocampal lesions have been linked to impairments in PI in animals^83,84^ and humans (e.g., temporal lobectomy^85^. In humans, reduced hippocampal volume has also been associated with deficits in tracking movement in a loop and estimating the rotation angle relative to a home location^25^. In addition, Wolbers and colleagues^27^ found a positive correlation between accuracy in pointing to a starting location after walking two legs of a triangle and activation in the right hippocampus. This is consistent with research showing that activity in the left and right hippocampus^26^ and the posterior hippocampus^86^ increased with PI performance.

The negative relationship between mapping performance and hippocampal volume may initially seem counterintuitive, given that superior spatial abilities are typically associated with larger hippocampal volumes. However, this pattern likely reflects the fact that mapping performance in SPACE can be supported by both hippocampal-dependent and hippocampal-independent processes^87,88^. Prior work^32,46,89^ has shown that participants can solve spatial tasks using either place-based strategies that recruit the hippocampus or response-based strategies that rely more heavily on the caudate (although we found no direct association between caudate volume and SPACE performance; Supplementary Table 4). As such, mapping accuracy alone may conflate hippocampal-dependent reconstruction with alternative, compensatory strategies, particularly in older adults, leading to a statistically robust but theoretically misleading association with hippocampal volume. By contrast, the multiplicative PI × mapping term isolates the component of performance in which accurate encoding during PI and accurate reconstruction during mapping co-occur. As demonstrated in Supplementary Information 5, this joint metric shows a monotonic relationship with hippocampal volume that is not apparent when either task is considered in isolation. Although modelling a product term without accompanying main effects is less common, regression theory recognises that such terms may be meaningfully interpreted as standalone predictors when they represent the theoretically relevant quantity of interest rather than a moderation effect^90^.

In our study, neither the pointing error nor the interaction between PI distance error and pointing error significantly predicted hippocampal volume. Previous studies reported mixed findings on the relationship between pointing performance and hippocampal volume^39,53^. A possible explanation for these contradictory findings may lie in differences in the environment and the pointing task employed. Indeed, Schinazi and colleagues^39^ found that performance on an off-site pointing task that relied on allocentric knowledge was associated with hippocampal volume after participants learned the locations of landmarks in a real-world setting. In the off-site pointing task, participants were blindfolded, disoriented, and taken to a testing room, where they performed judgments of relative direction while still blindfolded. Specifically, participants were required to mentally visualise their position and facing direction before pointing (e.g., “Imagine you are standing in front of building X, facing building Y, now point to building Z”). In contrast, the on-site pointing task used in the VEs by Weisberg and colleagues^53^ and SPACE did not correlate with hippocampal volume. Here, successfully completing the on-site pointing test does not necessarily require allocentric knowledge, since participants are automatically positioned in front of each landmark. As such, on-site pointing can be performed using a mixture of transient egocentric (online) and enduring allocentric (offline) spatial representations^39^ supported by distinct neural systems^91,92^.

Performance in the perspective taking task in SPACE was also not associated with hippocampal volume. Here, the map provided during the perspective taking task in SPACE meant that participants did not need to rely on their memory of the landmarks’ positions in the VE acquired during the PI task. Although the offsite pointing task in Schinazi and colleagues’ study^39^ and the perspective taking task in SPACE rely on allocentric knowledge, only the offsite pointing task required participants to learn and build a cognitive map of their environment. Because the perspective taking task in SPACE is solved using the externally provided map rather than relying on internally encoded landmark representations acquired during PI, we did not examine a combined PI × perspective taking interaction. Such a combination would not capture the same synergy as other downstream navigation tasks (i.e., pointing and mapping), which depend on the accurate encoding and subsequent reuse of landmark representations.

Our regression models also evaluated whether paper-and-pencil neuropsychological tests typically administered to screen for cognitive impairment were associated with hippocampal volume. At the feature selection stage, none of the tests in the neuropsychological battery (i.e., Maze Task, D-CAT, TMT-A, TMT-B, Dual-Task) showed a significant association. There is limited research on the relationship between neuropsychological test scores and hippocampal volume in healthy and cognitively impaired patients. To our knowledge, only a few studies have found that performance on the TMT-B is moderately correlated with hippocampal volume in healthy^93^ and non-demented patients^94^. However, this relationship could also be largely explained by differences in age, sex and education^94^. Previous studies have found a link between lower total MoCA scores^61–66^ or MoCA subdomains^60,64–66^ and hippocampal atrophy in healthy and cognitively impaired patients. Notably, neither the MoCA total score nor its visuospatial component was related to hippocampal volume in our study. Instead, only the naming and orientation subdomains showed small to modest associations with hippocampal volume, although the overall model fit for the MoCA subdomain model was significant and modest. These findings diverge from the stronger visuospatial associations reported by Gupta and colleagues^66^, while showing partial convergence with the naming-related association observed by Paul and colleagues^60^. More importantly, these results support the view that the navigation tasks in SPACE may complement standard neuropsychological screening by capturing hippocampal-dependent processes that are not adequately indexed by the MoCA or brief paper-and-pencil tests.

Despite extensive evidence demonstrating that the entorhinal cortex is implicated in navigation^13,14,23,95–97^, our feature selection analyses revealed no association between the tasks in SPACE and entorhinal cortex volume. Among candidate predictor tests across the models, only age and TMT-A performance showed a small association with entorhinal cortex volume. Although atrophy of the entorhinal cortex has been previously associated with memory decline in healthy adults^98^, it has not been clearly shown to predict spatial navigation performance^99^. In contrast to our results, previous studies reported that a smaller entorhinal cortex volume was associated with poorer navigation abilities, as measured using the Santa Barbara Sense of Direction Scale (SBSOD)^100^, and with greater errors in an immersive virtual reality PI test^31^. However, both studies included patients diagnosed with cognitive impairment.

While this study offers valuable insights into the role SPACE can play in assessing hippocampal structural integrity, a few limitations are worth noting. Firstly, due to known gender differences in spatial performance and strategy use, we deliberately recruited male participants. Research in spatial cognition has consistently shown that gender influences navigation performance across both self-report and behavioural measures^101–104^. These differences also extend to navigation strategy use^105,106^, confidence^107^, spatial anxiety^108^, and sensitivity to task constraints such as time pressure^109,110^. Importantly, these effects vary with task demands and environmental cues^111–113^, as well as cultural context^105^, making gender a non-trivial confound in navigation research. More recently, a large-scale study found that gender differences in navigation are also influenced by societal gender equity, with larger performance gaps observed in countries with lower gender equality^114^. Although Singapore, where this cohort was recruited, ranks relatively high in the Global Gender Gap Index^115^ within Southeast Asia (gender equality score = 75%), it is only mid-ranked globally (47th of 148 countries), suggesting that gender-related performance differences may still be present in this sample. Accordingly, to minimise behavioural heterogeneity and maximise statistical power in this initial proof-of-concept study, we adopted a homogeneous male sample. This choice was methodological and does not imply that SPACE is intended to be gender specific. Our broader research programme using SPACE includes ongoing and planned studies with mixed-gender samples^67,69,70^ that will establish gender-specific performance norms and examine whether the association between navigation behaviour and hippocampal integrity differs across genders.

Secondly, it is possible that performance on some of the SPACE tasks was influenced by age-related factors beyond spatial ability, including usability challenges related to visual status and familiarity with digital interfaces^116–123^. SPACE already incorporates enlarged icons and a user-friendly interface designed to support accessibility across age groups. Prior to the present study, SPACE underwent extensive usability testing with young, middle-aged, and older adults^67^, and no participants reported difficulties with landmark visibility or discriminability. Additionally, all participants in the current study completed a comprehensive training phase before performing the tasks in SPACE and received step-by-step instructions for each task. Although no objective measures of visual acuity were collected, the health and demographics questionnaire included a question about the presence of visual defects, and participants were instructed to complete the assessment with their glasses or contact lenses. Regression analyses indicated that self-reported visual defects were not associated with performance in SPACE tasks (Supplementary Table 8). We also explicitly assessed participants’ prior experience with tablet devices and examined their influence on performance across all SPACE tasks while controlling for age and education. Similar to visual defects, tablet experience did not emerge as a significant predictor in any of the models (Supplementary Table 9).

Finally, the cross-sectional design of the present study and the modest sample size warrant careful interpretation of the findings. The cohort comprised healthy older adults aged 55–79 years, which limits the generalisability of the results to other age groups and precludes inferences about longitudinal changes in hippocampal atrophy. Although the sample size (*N* = 40) is comparable to prior neuroimaging studies of spatial navigation^25,36,37,39,46,47,54,89,124^, the use of regression models with multiple predictors in a limited sample increases the risk of overfitting and model instability. To mitigate this concern, we conducted comprehensive regression diagnostics, which indicated that the model assumptions were met (Supplementary Information 6). We also repeated the analyses using models with fewer predictors (Supplementary Tables 5–7), which yielded a consistent pattern of results. Finally, post hoc power estimates indicated that the feature selection and hierarchical regression models involving SPACE measures were characterised by large effect sizes (Cohen’s f² ≥ 0.87). Future longitudinal work in larger, more diverse samples will be necessary to determine how navigation performance relates to changes in the hippocampus across the ageing continuum and in pathological ageing.

## Conclusion

In conclusion, our findings revealed that performance in SPACE is associated with hippocampal volume, beyond age, education, and commonly used neuropsychological tests, in a sample of older male adults. Critically, participants who successfully completed both the PI and the mapping tasks had larger hippocampal volumes. These findings highlight some limitations of traditional neuropsychological assessments, which primarily target memory and attention and may insufficiently capture spatial navigation processes that are closely linked to hippocampal integrity. Incorporating spatial navigation tests into neuropsychological batteries may therefore enhance sensitivity to hippocampal structural differences, particularly in non-clinical older adult populations. Altogether, SPACE has potential as a non-invasive, scalable, and cost-effective tool to complement existing cognitive assessments, though further validation in larger, clinically diverse samples is warranted.

## Methods

### Participants

We recruited 40 male participants from the community and the *Lions Befrienders Service Association*, aged 55–79 (mean = 67, SD = 6). Given the large gender differences in navigation performance and strategy use^54,102–104^, we deliberately recruited only male participants to reduce possible confounds in our analysis. An a priori power analysis conducted using GPower (linear multiple regression, R² increase in a hierarchical model) indicated that 40 participants would be required to achieve 80% power at α = 0.05 to detect a large incremental effect (*f²* = 0.35) for the second step of the model (four SPACE predictors entered after age and education). For each step of the hierarchical model, we also calculated the achieved power, which is presented together with the model fit for significant models. To be eligible for the study, participants were screened for a decline in cognitively relevant functional abilities using the Everyday Cognition Scale, with a cut-off score below one^125^. Participants with any physical disability, significant neurological disease, or contraindications to MRI were deemed ineligible. Written informed consent was obtained in the participants’ preferred language before any research procedure started. The ethics approval was obtained from the National University of Singapore Institutional Review Board (NUS-IRB Reference Code: NUS-IRB-2022-466). All procedures adhered to the Declaration of Helsinki.

## Materials

### Sociodemographic, Navigation, and health measurements

As part of a larger set of studies, the sociodemographic, navigation and health questionnaire collected information on age, ethnicity, education, profession, handedness, tablet experience, previous navigation training, and sense of direction (Santa Barbara Sense of Direction^126^. The questionnaire also included questions on their health status, such as visual defects, chronic conditions, history of traumatic brain injury, depression, anxiety, and stress (DASS-21^127^). Additionally, the questionnaire addressed health habits, such as diet, smoking, alcohol consumption, falls in the past year, daily hours of sleep, and weekly hours of walking and vigorous physical activity. Since the paper’s primary focus is predicting hippocampal volume from SPACE performance, data collected from the navigation and health questionnaires were excluded from the analysis and reserved for subsequent publications.

### Neuropsychological tests

The participants’ cognitive abilities were assessed using the MoCA, Maze Task, D-CAT, TMT, and Dual-Task.

*MoCA*. The MoCA is a widely used screening tool for detecting cognitive impairment with a sensitivity and specificity of 90% and 87%, respectively^56^. The MoCA evaluates six cognitive domains: memory, executive function, visuospatial, language, attention, and orientation. Administering the MoCA takes approximately 15 min. A score of 25 or lower indicates MCI.

*Maze Task*. The Maze Task was used to assess cognitive abilities related to spatial and visual perception^128^. In the test, participants are presented with a maze on a sheet of paper and asked to find the way out by drawing a line from the entrance to the exit as quickly as possible. The outcome variable is the time required to complete the task, with shorter times indicating better performance.

*Digit Cancellation Test (D-CAT)*. The D-CAT was developed to measure attention^128^. In this task, participants are required to cross out the target digits printed on a page interspersed with other numbers within 45 seconds. The final score is calculated as the subtraction of the number of incorrectly cancelled digits from the total number of correctly cancelled digits. The higher the final score, the better the performance.

*Trail Making Test (TMT)*. The TMT was used to measure attention, visual screening ability, and processing speed^129^. The test consists of two parts. In the first part (TMT–A), participants are asked to connect circles with numbers in ascending order. In the second part (TMT–B), participants have to connect the circles by switching between numbers and letters in consecutive order (e.g., 1, A, 2, B). The time to completion in seconds was reported separately for each part of the TMT test. A shorter time indicates better performance.

*Dual-Task*. The Dual-Task test assessed the ability to perform two tasks concurrently. This task consisted of performing digit recall and tracking tasks separately, then simultaneously^130,131^. First, each participant underwent a digit span assessment to determine their maximum digit span capacity. This was followed by two trials involving digit recall and tracking tasks to familiarise the participants with the Dual-Task test. For the digit recall task, participants are presented with a list of numbers and are required to verbally repeat them in the exact order they are read. For the tracking task, participants trace the paper with a pencil, joining all the circles along a predefined route as quickly as possible. Each task was restricted to 1.5 minutes. After familiarisation, the Dual-Task test was administered with the same time limit, and subsequently, participants were required to complete both tasks simultaneously. The final performance score is computed as the combined proportional performance across both tasks^130^.

### Spatial performance assessment for cognitive evaluation (SPACE)

SPACE is a novel iPad-based digital assessment designed to assess spatial navigation deficits indicative of cognitive impairment^67,70^. Table 6 provides a description of each task in SPACE.

**Table 6:** The tasks in SPACE.

Visuospatial training	Participants learn to rotate, translate, and combine these movements by navigating around the VE. Performance is quantified by measuring the time (in seconds) required for each player to complete all phases of the training.
**Path Integration (PI)**	Participants follow the robot from the rocket to two landmarks by walking along two sides of a triangle. At each landmark, the robot scans a different element that will be recalled in a later task. Participants are asked to return unguided to the rocket’s original position, completing the third side of the triangle. At the beginning of each trial, the rocket takes off and stays invisible until participants signal its landing after completing the trial. Performance is quantified by measuring the PI distance error, defined as the average distance between the player’s final position and the rocket’s original position for all PI trials.
**Pointing**	Participants perform a series of pointing judgments from one landmark to other landmarks encountered during the PI task. Performance is quantified by measuring the egocentric pointing error, defined as the average angular deviation (in degrees) from a starting location to the target location.
**Mapping**	Participants are asked to recreate the configuration of landmarks they learned in the PI task by dragging and dropping icons representing the landmarks. Performance is quantified by measuring mapping accuracy, computed using bidimensional regression^132,133^. Bidimensional regression assesses the degree of association (r^2^) between the correct map and the map built by the participant.
**Associative memory**	Participants are presented with a corrected top-down map of the landmarks and are asked to drag and drop icons representing the corresponding elements scanned by the robot during the PI task. Performance is quantified by measuring the associative memory score, computed as the percentage of correct pairings between scanned elements and landmarks. The associative memory task was excluded from all our analyses because of ceiling effects, with 90% of participants achieving a perfect score.
**Perspective taking**	Participants are provided with the correct top-down map of the environment and are asked to identify the direction of a landmark by imagining themselves standing at a specified location and facing a given orientation (e.g., "imagine you are standing at the tree, facing the waterfall, point to the rocket). Performance is quantified by measuring the perspective taking error, defined as the average angular deviation (in degrees) between the estimate made by the player and the target landmark for all trials in the task.

**Fig. 3:**
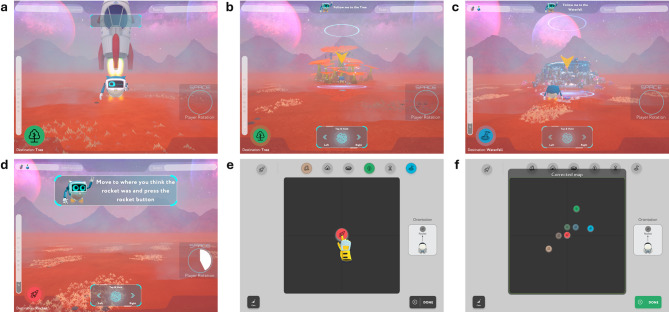
Tasks in SPACE. Screenshots of the PI (a-d) and mapping (e-f) tasks in SPACE. (a) The rocket takes off at the start of a PI trial. (b) The player follows a robot to the first landmark (e.g., Tree). (c) The player follows a robot to the second landmark (e.g., Waterfall). (d) The player estimates the orientation and distance from the second landmark back to the rocket. (e) The player drags and drops the icons of the landmarks to create a map of the environment. (f) An animation showing the correct positions of the landmarks is displayed.

Before each task, participants are presented with video instructions and receive real-time guidance as they progress through the assessment. SPACE includes visuospatial training, PI, and pointing tasks from a first-person perspective. The mapping, associative memory and perspective taking tasks are completed from a top-down perspective (Fig. 3).

### Image acquisition and processing

 Magnetic Resonance Imaging (MRI) was performed at the Singapore Centre for Translational MR Research using a 3 T Prisma-Fit scanner (Siemens Healthineers, Erlangen, Germany). The standardised neuroimaging protocol used in this study included 3D T1-weighted images (TR = 2300 ms, TE = 1.96 ms, TI = 900 ms, flip angle = 9॰, voxel size = 1 × 1 × 1 mm^3^). Structural T1-weighted image segmentation was conducted using Freesurfer version 7.4.1 (https://surfer.nmr.mgh.harvard.edu/). Hippocampus volumes were segmented using Freesurfer Hippocampus Subfield pipeline (https://surfer.nmr.mgh.harvard.edu/fswiki/ HippocampalSubfieldsAndNucleiOfAmygdala). Brain volumetric data were further corrected with the total intracranial volume ratio obtained from the segmentation. Quality check was conducted on the brain mask output from Freesurfer, and those with segmentation errors were manually corrected for the remaining segmentation steps used in Freesurfer’s “recon-all” command.

### Design and analysis

Before conducting inferential statistics, we verified that our data met the assumptions of linear regression. Across all models, diagnostic checks of residual normality, homoscedasticity, independence, and multicollinearity were satisfied, with no observations exerting disproportionate influence on the fitted models (see Supplementary Information 6). To identify which components of the MoCA, the neuropsychological battery and SPACE were associated with hippocampal and entorhinal cortex volume, we first conducted a series of feature selection analyses. Separate linear regression models were fitted for the left and right hemispheres of each region, with age and education included as covariates to control for demographic variance. Within these models, candidate predictors were entered simultaneously to determine which variables were independently associated with regional volume. Because performance in the pointing and mapping task depends on accurate spatial encoding during the PI task, we additionally tested a composite spatial efficiency measure defined as the product (interaction term) of PI distance error × pointing error and PI distance error × mapping performance. This multiplicative term was treated as a single predictor capturing joint encoding–reconstruction efficiency rather than as a moderation^90^ effect and was evaluated in separate regression models alongside age and education. No composite term was tested for the perspective taking task, given that this task could be solved using an externally provided map and did not depend on internally encoded landmark representations acquired during PI. Variables identified as significant in the feature selection analyses were subsequently entered into hierarchical regression models. In these hierarchical models, age and education were entered in the first block, followed by the selected predictors from the cognitive tests and SPACE in subsequent blocks.

For each regression model, we report the F-statistic and overall model fit (R², adjusted R²) along with unstandardised regression coefficients (Est.), standard errors, and p-values in the tables. In the text, we additionally report standardised regression coefficients (β) for predictors that made a statistically significant unique contribution. Model comparisons in the hierarchical regressions were conducted using F-tests and changes in explained variance (ΔR²). All statistical analyses were performed using JAMOVI version 2.3.28, SPSS version 29, and RStudio version 4.2.2. Statistical significance was set at *p* < 0.05.

## Supplementary Information

Below is the link to the electronic supplementary material.

Supplementary Material 1

## Data Availability

Due to the sensitive nature of the data, access to the datasets supporting the findings of this study can be obtained from the corresponding author upon reasonable request and following ethics approval.

## Abbreviations

AD: Alzheimer’s disease
D-CAT: Digit cancellation test
MRI: Magnetic resonance imaging
MoCA: Montreal cognitive assessment
PI: Path integration
SPACE: Spatial performance assessment for cognitive evaluation
TMT: Trail making test
VE: Virtual environments
MCI: Mild Cognitive Impairment

## References

[1] O’Shea, A., Cohen, R. A., Porges, E. C., Nissim, N. R. & Woods, A. J. Cognitive aging and the hippocampus in older adults. *Front. Aging Neurosci.* **8**, 298. 10.3389/fnagi.2016.00298 (2016). 28008314 10.3389/fnagi.2016.00298 PMC5143675

[2] Bettio, L. E. B., Rajendran, L. & Gil-Mohapel, J. The effects of aging in the hippocampus and cognitive decline. *Neurosci. Biobehav Rev.* **79**, 66–86. 10.1016/j.neubiorev.2017.04.030 (2017). 28476525 10.1016/j.neubiorev.2017.04.030

[3] Jernigan, T. L. et al. Cerebral structure on MRI, part I: localization of age-related changes. *Biol. Psychiatry*. **29**, 55–67. 10.1016/0006-3223(91)90210-d (1991). 2001446 10.1016/0006-3223(91)90210-d

[4] Golomb, J. et al. Hippocampal atrophy in normal aging. An association with recent memory impairment. *Arch. Neurol.* **50**, 967–973. 10.1001/archneur.1993.00540090066012 (1993). 8363451 10.1001/archneur.1993.00540090066012

[5] Persson, J. et al. Structure-function correlates of cognitive decline in aging. *Cereb. Cortex*. **16**, 907–915. 10.1093/cercor/bhj036 (2006). 16162855 10.1093/cercor/bhj036

[6] Barnes, J. et al. A meta-analysis of hippocampal atrophy rates in alzheimer’s disease. *Neurobiol. Aging*. **30**, 1711–1723. 10.1016/j.neurobiolaging.2008.01.010 (2009). 18346820 10.1016/j.neurobiolaging.2008.01.010 PMC2773132

[7] Buckner, R. L. Functional-anatomic correlates of control processes in memory. *J. Neurosci.* **23**, 3999–4004. 10.1523/JNEUROSCI.23-10-03999.2003 (2003). 12764084 10.1523/JNEUROSCI.23-10-03999.2003 PMC6741083

[8] Squire, L. R. & Zola-Morgan, S. The medial Temporal lobe memory system. *Science* **253**, 1380–1386. 10.1126/science.1896849 (1991). 1896849 10.1126/science.1896849

[9] Eichenbaum, H., Yonelinas, A. P. & Ranganath, C. The medial Temporal lobe and recognition memory. *Annu. Rev. Neurosci.* **30**, 123–152. 10.1146/annurev.neuro.30.051606.094328 (2007). 17417939 10.1146/annurev.neuro.30.051606.094328 PMC2064941

[10] Ruiz, N. A., Meager, M. R., Agarwal, S. & Aly, M. The medial Temporal lobe is critical for Spatial relational perception. *J. Cogn. Neurosci.* **32**, 1780–1795. 10.1162/jocn_a_01583 (2020). 32427068 10.1162/jocn_a_01583

[11] O’Keefe, J., Burgess, N., Donnett, J. G., Jeffery, K. J. & Maguire, E. A. Place cells, navigational accuracy, and the human hippocampus. *Philos. Trans. R Soc. Lond. B Biol. Sci.* **353**, 1333–1340. 10.1098/rstb.1998.0287 (1998). 9770226 10.1098/rstb.1998.0287 PMC1692339

[12] Rolls, E. T. Hippocampal Spatial view cells for memory and navigation, and their underlying connectivity in humans. *Hippocampus* **33**, 533–572. 10.1002/hipo.23467 (2023). 36070199 10.1002/hipo.23467 PMC10946493

[13] Hafting, T., Fyhn, M., Molden, S. & Moser, M. B. Moser, E. I. Microstructure of a Spatial map in the entorhinal cortex. *Nature* **436**, 801–806. 10.1038/nature03721 (2005). 15965463 10.1038/nature03721

[14] Jacobs, J. et al. Direct recordings of grid-like neuronal activity in human Spatial navigation. *Nat. Neurosci.* **16**, 1188–1190. 10.1038/nn.3466 (2013). 23912946 10.1038/nn.3466 PMC3767317

[15] Hassabis, D. et al. Decoding neuronal ensembles in the human hippocampus. *Curr. Biol.* **19**, 546–554. 10.1016/j.cub.2009.02.033 (2009). 19285400 10.1016/j.cub.2009.02.033 PMC2670980

[16] Ekstrom, A. D. et al. Cellular networks underlying human Spatial navigation. *Nature* **425**, 184–188. 10.1038/nature01964 (2003). 12968182 10.1038/nature01964

[17] Schinazi, V. R. & Thrash, T. Cognitive neuroscience of Spatial and geographic thinking. *Handb. Behav. Cogn. Geogr.* 154–174. 10.4337/9781784717544.00016 (2018).

[18] Epstein, R. A., Patai, E. Z., Julian, J. B. & Spiers, H. J. The cognitive map in humans: Spatial navigation and beyond. *Nat. Neurosci.* **20**, 1504–1513. 10.1038/nn.4656 (2017). 29073650 10.1038/nn.4656 PMC6028313

[19] Maguire, E. A., Frackowiak, R. S. & Frith, C. D. Recalling routes around london: activation of the right hippocampus in taxi drivers. *J. Neurosci.* **17**, 7103–7110. 10.1523/JNEUROSCI.17-18-07103.1997 (1997). 9278544 10.1523/JNEUROSCI.17-18-07103.1997 PMC6573257

[20] Maguire, E. A. et al. Knowing where and getting there: a human navigation network. *Science* **280**, 921–924. 10.1126/science.280.5365.921 (1998). 9572740 10.1126/science.280.5365.921

[21] Wolbers, T. & Buchel, C. Dissociable retrosplenial and hippocampal contributions to successful formation of survey representations. *J. Neurosci.* **25**, 3333–3340. 10.1523/JNEUROSCI.4705-04.2005 (2005). 15800188 10.1523/JNEUROSCI.4705-04.2005 PMC6724902

[22] Morgan, L. K., Macevoy, S. P., Aguirre, G. K. & Epstein, R. A. Distances between real-world locations are represented in the human hippocampus. *J. Neurosci.* **31**, 1238–1245. 10.1523/JNEUROSCI.4667-10.2011 (2011). 21273408 10.1523/JNEUROSCI.4667-10.2011 PMC3074276

[23] Howard, L. R. et al. The hippocampus and entorhinal cortex encode the path and Euclidean distances to goals during navigation. *Curr. Biol.* **24**, 1331–1340. 10.1016/j.cub.2014.05.001 (2014). 24909328 10.1016/j.cub.2014.05.001 PMC4062938

[24] Patai, E. Z. et al. Hippocampal and retrosplenial goal distance coding after Long-term consolidation of a Real-World environment. *Cereb. Cortex*. **29**, 2748–2758. 10.1093/cercor/bhz044 (2019). 30916744 10.1093/cercor/bhz044 PMC6519689

[25] Chrastil, E. R., Sherrill, K. R., Aselcioglu, I., Hasselmo, M. E. & Stern, C. E. Individual Differences in Human Path Integration Abilities Correlate with Gray Matter Volume in Retrosplenial Cortex, Hippocampus, and Medial Prefrontal Cortex. *eNeuro* 4 (2017). 10.1523/ENEURO.0346-16.2017 10.1523/ENEURO.0346-16.2017 PMC5392707 28451633

[26] Chrastil, E. R., Sherrill, K. R., Hasselmo, M. E. & Stern, C. E. There and back again: hippocampus and retrosplenial cortex track homing distance during human path integration. *J. Neurosci.* **35**, 15442–15452. 10.1523/JNEUROSCI.1209-15.2015 (2015). 26586830 10.1523/JNEUROSCI.1209-15.2015 PMC6605486

[27] Wolbers, T., Wiener, J. M., Mallot, H. A. & Buchel, C. Differential recruitment of the hippocampus, medial prefrontal cortex, and the human motion complex during path integration in humans. *J. Neurosci.* **27**, 9408–9416. 10.1523/JNEUROSCI.2146-07.2007 (2007). 17728454 10.1523/JNEUROSCI.2146-07.2007 PMC6673121

[28] Shrager, Y., Kirwan, C. B. & Squire, L. R. Neural basis of the cognitive map: path integration does not require hippocampus or entorhinal cortex. *Proc. Natl. Acad. Sci. U S A*. **105**, 12034–12038. 10.1073/pnas.0805414105 (2008). 18687893 10.1073/pnas.0805414105 PMC2575247

[29] Kim, S., Sapiurka, M., Clark, R. E. & Squire, L. R. Contrasting effects on path integration after hippocampal damage in humans and rats. *Proc. Natl. Acad. Sci. U S A*. **110**, 4732–4737. 10.1073/pnas.1300869110 (2013). 23404706 10.1073/pnas.1300869110 PMC3606992

[30] Segen, V., Ying, J., Morgan, E., Brandon, M. & Wolbers, T. Path integration in normal aging and alzheimer’s disease. *Trends Cogn. Sci.* **26**, 142–158. 10.1016/j.tics.2021.11.001 (2022). 34872838 10.1016/j.tics.2021.11.001

[31] Howett, D. et al. Differentiation of mild cognitive impairment using an entorhinal cortex-based test of virtual reality navigation. *Brain* **142**, 1751–1766. 10.1093/brain/awz116 (2019). 31121601 10.1093/brain/awz116 PMC6536917

[32] Iaria, G., Petrides, M., Dagher, A., Pike, B. & Bohbot, V. D. Cognitive strategies dependent on the hippocampus and caudate nucleus in human navigation: variability and change with practice. *J. Neurosci.* **23**, 5945–5952. 10.1523/JNEUROSCI.23-13-05945.2003 (2003). 12843299 10.1523/JNEUROSCI.23-13-05945.2003 PMC6741255

[33] Marchette, S. A., Bakker, A. & Shelton, A. L. Cognitive mappers to creatures of habit: differential engagement of place and response learning mechanisms predicts human navigational behavior. *J. Neurosci.* **31**, 15264–15268. 10.1523/jneurosci.3634-11.2011 (2011). 22031872 10.1523/JNEUROSCI.3634-11.2011 PMC4826051

[34] Arnold, A. E., Burles, F., Bray, S., Levy, R. M. & Iaria, G. Differential neural network configuration during human path integration. *Front. Hum. Neurosci.* **8**, 263. 10.3389/fnhum.2014.00263 (2014). 24808849 10.3389/fnhum.2014.00263 PMC4010772

[35] Spiers, H. J. & Gilbert, S. J. Solving the detour problem in navigation: a model of prefrontal and hippocampal interactions. *Front. Hum. Neurosci.* **9**, 125. 10.3389/fnhum.2015.00125 (2015). 25852515 10.3389/fnhum.2015.00125 PMC4366647

[36] Maguire, E. A. et al. Navigation-related structural change in the hippocampi of taxi drivers. *Proc. Natl. Acad. Sci. U S A*. **97**, 4398–4403. 10.1073/pnas.070039597 (2000). 10716738 10.1073/pnas.070039597 PMC18253

[37] Maguire, E. A., Woollett, K. & Spiers, H. J. London taxi drivers and bus drivers: a structural MRI and neuropsychological analysis. *Hippocampus* **16**, 1091–1101. 10.1002/hipo.20233 (2006). 17024677 10.1002/hipo.20233

[38] Woollett, K. & Maguire, E. A. Acquiring the knowledge of london’s layout drives structural brain changes. *Curr. Biol.* **21**, 2109–2114. 10.1016/j.cub.2011.11.018 (2011). 22169537 10.1016/j.cub.2011.11.018 PMC3268356

[39] Schinazi, V. R., Nardi, D., Newcombe, N. S., Shipley, T. F. & Epstein, R. A. Hippocampal size predicts rapid learning of a cognitive map in humans. *Hippocampus* **23**, 515–528. 10.1002/hipo.22111 (2013). 23505031 10.1002/hipo.22111 PMC3690629

[40] Brown, T. I., Whiteman, A. S., Aselcioglu, I. & Stern, C. E. Structural differences in hippocampal and prefrontal Gray matter volume support flexible context-dependent navigation ability. *J. Neurosci.* **34**, 2314–2320. 10.1523/JNEUROSCI.2202-13.2014 (2014). 24501370 10.1523/JNEUROSCI.2202-13.2014 PMC3913873

[41] Ezzati, A. et al. Differential association of left and right hippocampal volumes with verbal episodic and Spatial memory in older adults. *Neuropsychologia* **93**, 380–385. 10.1016/j.neuropsychologia.2016.08.016 (2016). 27542320 10.1016/j.neuropsychologia.2016.08.016 PMC5154822

[42] Chen, K. H., Chuah, L. Y., Sim, S. K. & Chee, M. W. Hippocampal region-specific contributions to memory performance in normal elderly. *Brain Cogn.* **72**, 400–407. 10.1016/j.bandc.2009.11.007 (2010). 20044193 10.1016/j.bandc.2009.11.007

[43] Head, D. & Isom, M. Age effects on wayfinding and route learning skills. *Behav. Brain Res.* **209**, 49–58. 10.1016/j.bbr.2010.01.012 (2010). 20085784 10.1016/j.bbr.2010.01.012

[44] Driscoll, I. et al. The aging hippocampus: cognitive, biochemical and structural findings. *Cereb. Cortex*. **13**, 1344–1351. 10.1093/cercor/bhg081 (2003). 14615299 10.1093/cercor/bhg081

[45] Korthauer, L. E. et al. Correlates of virtual navigation performance in older adults. *Neurobiol. Aging*. **39**, 118–127. 10.1016/j.neurobiolaging.2015.12.003 (2016). 26923408 10.1016/j.neurobiolaging.2015.12.003 PMC4773923

[46] Konishi, K. & Bohbot, V. D. Spatial navigational strategies correlate with Gray matter in the hippocampus of healthy older adults tested in a virtual maze. *Front. Aging Neurosci.* **5**, 1. 10.3389/fnagi.2013.00001 (2013). 23430962 10.3389/fnagi.2013.00001 PMC3576603

[47] Sodums, D. J. & Bohbot, V. D. Negative correlation between grey matter in the hippocampus and caudate nucleus in healthy aging. *Hippocampus* **30**, 892–908. 10.1002/hipo.23210 (2020). 32384195 10.1002/hipo.23210

[48] de Toledo-Morrell, L. et al. Hemispheric differences in hippocampal volume predict verbal and Spatial memory performance in patients with alzheimer’s disease. *Hippocampus* **10**, 136–142. 10.1002/(SICI)1098-1063(2000)10:2%3C136 (2000). :AID-HIPO2>3.0.CO;2-J 10791835 10.1002/(SICI)1098-1063(2000)10:2<136::AID-HIPO2>3.0.CO;2-J

[49] Nedelska, Z. et al. Spatial navigation impairment is proportional to right hippocampal volume. *Proc. Natl. Acad. Sci. U S A*. **109**, 2590–2594. 10.1073/pnas.1121588109 (2012). 22308496 10.1073/pnas.1121588109 PMC3289383

[50] deIpolyi, A. R., Rankin, K. P., Mucke, L., Miller, B. L. & Gorno-Tempini, M. L. Spatial cognition and the human navigation network in AD and MCI. *Neurology* **69**, 986–997. 10.1212/01.wnl.0000271376.19515.c6 (2007). 17785667 10.1212/01.wnl.0000271376.19515.c6

[51] Maguire, E. A. et al. Navigation expertise and the human hippocampus: a structural brain imaging analysis. *Hippocampus* **13**, 250–259. 10.1002/hipo.10087 (2003). 12699332 10.1002/hipo.10087

[52] Clark, I. A. et al. Does hippocampal volume explain performance differences on hippocampal-dependant tasks? *Neuroimage* 221, 117211 (2020). 10.1016/j.neuroimage.2020.117211 10.1016/j.neuroimage.2020.117211 PMC7762813 32739555

[53] Weisberg, S. M., Newcombe, N. S. & Chatterjee, A. Everyday taxi drivers: do better navigators have larger hippocampi? *Cortex* **115**, 280–293. 10.1016/j.cortex.2018.12.024 (2019). 30884282 10.1016/j.cortex.2018.12.024 PMC6513697

[54] Alina, S. et al. Chrastil. Do total hippocampus and hippocampal subfield volumes relate to navigation ability? A call towards methodological consistency. *Cortex*10.1016/j.cortex.2024.08.011 (2024). 10.1016/j.cortex.2024.08.011 39566126

[55] He, Q. & Brown, T. I. Heterogeneous correlations between hippocampus volume and cognitive map accuracy among healthy young adults. *Cortex* **124**, 167–175. 10.1016/j.cortex.2019.11.011 (2020). 31901562 10.1016/j.cortex.2019.11.011 PMC7069601

[56] Nasreddine, Z. S. et al. The Montreal cognitive Assessment, moca: a brief screening tool for mild cognitive impairment. *J. Am. Geriatr. Soc.* **53**, 695–699. 10.1111/j.1532-5415.2005.53221.x (2005). 15817019 10.1111/j.1532-5415.2005.53221.x

[57] Cheng, X. et al. Associations between brain structures, cognition and dual-task performance in patients with mild cognitive impairment: A study based on voxel-based morphology. *Hum. Mov. Sci.* **97**, 103257. 10.1016/j.humov.2024.103257 (2024). 39126810 10.1016/j.humov.2024.103257

[58] Gao, Y. et al. Changes of brain structure in parkinson’s disease patients with mild cognitive impairment analyzed via VBM technology. *Neurosci. Lett.* **658**, 121–132. 10.1016/j.neulet.2017.08.028 (2017). 28823894 10.1016/j.neulet.2017.08.028

[59] Del Brutto, O. H., Mera, R. M., Zambrano, M., Soriano, F. & Lama, J. Global cortical atrophy (GCA) associates with worse performance in the Montreal cognitive assessment (MoCA). A population-based study in community-dwelling elders living in rural Ecuador. *Arch. Gerontol. Geriatr.* **60**, 206–209. 10.1016/j.archger.2014.09.010 (2015). 25306507 10.1016/j.archger.2014.09.010

[60] Paul, R. et al. Neuroimaging signatures and cognitive correlates of the Montreal cognitive assessment screen in a nonclinical elderly sample. *Arch. Clin. Neuropsychol.* **26**, 454–460. 10.1093/arclin/acr017 (2011). 21642663 10.1093/arclin/acr017 PMC3142949

[61] Liang, L. et al. Structural and functional hippocampal changes in subjective cognitive decline from the community. *Front. Aging Neurosci.* **12**, 64. 10.3389/fnagi.2020.00064 (2020). 32256336 10.3389/fnagi.2020.00064 PMC7090024

[62] Liang, X. et al. The role of MRI biomarkers and their interactions with cognitive status and APOE epsilon4 in nondemented elderly subjects. *Neurodegener Dis.* **18**, 270–280. 10.1159/000495754 (2018). 30673663 10.1159/000495754

[63] Feng, Q. et al. Machine learning classifiers and associations of cognitive performance with hippocampal subfields in amnestic mild cognitive impairment. *Front. Aging Neurosci.* **15**, 1273658. 10.3389/fnagi.2023.1273658 (2023). 38099266 10.3389/fnagi.2023.1273658 PMC10719844

[64] Ritter, A., Hawley, N., Banks, S. J. & Miller, J. B. The association between Montreal cognitive assessment memory scores and hippocampal volume in a neurodegenerative disease sample. *J. Alzheimers Dis.* **58**, 695–699. 10.3233/JAD-161241 (2017). 28453481 10.3233/JAD-161241 PMC5467712

[65] Xiao, Y., Hu, Y. & Huang, K. Alzheimer’s disease Neuroimaging, I. Atrophy of hippocampal subfields relates to memory decline during the pathological progression of alzheimer’s disease. *Front. Aging Neurosci.* **15**, 1287122. 10.3389/fnagi.2023.1287122 (2023). 38149170 10.3389/fnagi.2023.1287122 PMC10749921

[66] Gupta, M. et al. Association of 3.0-T brain magnetic resonance imaging biomarkers with cognitive function in the Dallas heart study. *JAMA Neurol.* **72**, 170–175. 10.1001/jamaneurol.2014.3418 (2015). 25485570 10.1001/jamaneurol.2014.3418

[67] Colombo, G. et al. Detecting cognitive impairment through an age-friendly serious game: the development and usability of the Spatial performance assessment for cognitive evaluation (SPACE). *Comput. Hum. Behav.* **160**10.1016/j.chb.2024.108349 (2024).

[68] Tian, N., Colombo, G. & Schinazi, V. From play to detection: Mini-SPACE as a serious game for unsupervised Cognitive Impairment screening. *arXiv* https://doi.org/10.48550/arXiv.2511.12068 (2025),

[69] Colombo, G. et al. Spatial navigation as a digital marker for clinically differentiating cognitive impairment severity. *MedRxiv*10.1101/2025.06.05.25329079 (2025). 41787066 10.1038/s43856-026-01484-y PMC13086951

[70] Colombo, G. et al. Beyond Traditional Assessments of Cognitive Impairment: Exploring the Potential of Spatial Navigation Tasks. *MedRxiv* https://doi.org/10.1101/2024.10.12.24315402 (2024).

[71] Rana, A. K. et al. A comparison of measurement methods of hippocampal atrophy rate for predicting alzheimer’s dementia in the Aberdeen birth cohort of 1936. *Alzheimers Dement. (Amst)*. **6**, 31–39. 10.1016/j.dadm.2016.11.007 (2017). 28149941 10.1016/j.dadm.2016.11.007 PMC5266475

[72] Ezekiel, F. et al. Comparisons between global and focal brain atrophy rates in normal aging and alzheimer disease: boundary shift integral versus tracing of the entorhinal cortex and hippocampus. *Alzheimer Dis. Assoc. Disord*. **18**, 196–201 (2004). 15592130 PMC1820853

[73] Scahill, R. I. et al. A longitudinal study of brain volume changes in normal aging using serial registered magnetic resonance imaging. *Arch. Neurol.* **60**, 989–994. 10.1001/archneur.60.7.989 (2003). 12873856 10.1001/archneur.60.7.989

[74] Takeda, S. & Matsuzawa, T. Age-related brain atrophy: a study with computed tomography. *J. Gerontol.* **40**, 159–163. 10.1093/geronj/40.2.159 (1985). 3973356 10.1093/geronj/40.2.159

[75] Whitwell, J. L. et al. Rates of cerebral atrophy differ in different degenerative pathologies. *Brain* **130**, 1148–1158. 10.1093/brain/awm021 (2007). 17347250 10.1093/brain/awm021 PMC2752409

[76] Henneman, W. J. et al. Hippocampal atrophy rates in alzheimer disease: added value over whole brain volume measures. *Neurology* **72**, 999–1007. 10.1212/01.wnl.0000344568.09360.31 (2009). 19289740 10.1212/01.wnl.0000344568.09360.31 PMC2821835

[77] Duara, R. et al. Medial Temporal lobe atrophy on MRI scans and the diagnosis of alzheimer disease. *Neurology* **71**, 1986–1992. 10.1212/01.wnl.0000336925.79704.9f (2008). 19064880 10.1212/01.wnl.0000336925.79704.9f PMC2676975

[78] Noble, K. G. et al. Hippocampal volume varies with educational attainment across the life-span. *Front. Hum. Neurosci.* **6**, 307. 10.3389/fnhum.2012.00307 (2012). 23162453 10.3389/fnhum.2012.00307 PMC3494123

[79] Shpanskaya, K. S. et al. Educational attainment and hippocampal atrophy in the alzheimer’s disease neuroimaging initiative cohort. *J. Neuroradiol.* **41**, 350–357. 10.1016/j.neurad.2013.11.004 (2014). 24485897 10.1016/j.neurad.2013.11.004

[80] Sharp, E. S. & Gatz, M. Relationship between education and dementia: an updated systematic review. *Alzheimer Dis. Assoc. Disord*. **25**, 289–304. 10.1097/WAD.0b013e318211c83c (2011). 21750453 10.1097/WAD.0b013e318211c83c PMC3193875

[81] Devanand, D. P. et al. Hippocampal and entorhinal atrophy in mild cognitive impairment: prediction of alzheimer disease. *Neurology* **68**, 828–836. 10.1212/01.wnl.0000256697.20968.d7 (2007). 17353470 10.1212/01.wnl.0000256697.20968.d7

[82] Kim, H. B. et al. Modulation of associations between education years and cortical volume in alzheimer’s disease vulnerable brain regions by Abeta deposition and APOE epsilon4 carrier status in cognitively normal older adults. *Front. Aging Neurosci.* **15**, 1248531. 10.3389/fnagi.2023.1248531 (2023). 37829142 10.3389/fnagi.2023.1248531 PMC10565031

[83] Whishaw, I. Q., McKenna, J. E. & Maaswinkel, H. Hippocampal lesions and path integration. *Curr. Opin. Neurobiol.* **7**, 228–234. 10.1016/s0959-4388(97)80011-6 (1997). 9142750 10.1016/s0959-4388(97)80011-6

[84] McNaughton, B. L., Battaglia, F. P., Jensen, O., Moser, E. I. & Moser, M. B. Path integration and the neural basis of the ‘cognitive map’. *Nat. Rev. Neurosci.* **7**, 663–678. 10.1038/nrn1932 (2006). 16858394 10.1038/nrn1932

[85] Worsley, C. L. et al. Path integration following Temporal lobectomy in humans. *Neuropsychologia* **39**, 452–464. 10.1016/s0028-3932(00)00140-8 (2001). 11254927 10.1016/s0028-3932(00)00140-8

[86] Sherrill, K. R. et al. Hippocampus and retrosplenial cortex combine path integration signals for successful navigation. *J. Neurosci.* **33**, 19304–19313. 10.1523/JNEUROSCI.1825-13.2013 (2013). 24305826 10.1523/JNEUROSCI.1825-13.2013 PMC3850045

[87] Moffat, S. D., Kennedy, K. M., Rodrigue, K. M. & Raz, N. Extrahippocampal contributions to age differences in human Spatial navigation. *Cereb. Cortex*. **17**, 1274–1282. 10.1093/cercor/bhl036 (2007). 16857855 10.1093/cercor/bhl036

[88] Baumann, O. & Mattingley, J. B. Extrahippocampal contributions to Spatial navigation in humans: A review of the neuroimaging evidence. *Hippocampus* **31**, 640–657. 10.1002/hipo.23313 (2021). 33595156 10.1002/hipo.23313

[89] Bohbot, V. D., Lerch, J., Thorndycraft, B., Iaria, G. & Zijdenbos, A. P. Gray matter differences correlate with spontaneous strategies in a human virtual navigation task. *J. Neurosci.* **27**, 10078–10083. 10.1523/JNEUROSCI.1763-07.2007 (2007). 17881514 10.1523/JNEUROSCI.1763-07.2007 PMC6672675

[90] Cleves, M., Gould, W. & Guiererez, R. *G. An Introduction To Survival Analysis Using Stata* (Vol. Revised Edition (Stata, 2004).

[91] Burgess, N. Spatial memory: how egocentric and allocentric combine. *Trends Cogn. Sci.* **10**, 551–557. 10.1016/j.tics.2006.10.005 (2006). 17071127 10.1016/j.tics.2006.10.005

[92] Waller, D. & Hodgson, E. Transient and enduring Spatial representations under disorientation and self-rotation. *J. Exp. Psychol. Learn. Mem. Cogn.* **32**, 867–882. 10.1037/0278-7393.32.4.867 (2006). 16822154 10.1037/0278-7393.32.4.867 PMC1501085

[93] Duff, K. et al. Short-term repeat cognitive testing and its relationship to hippocampal volumes in older adults. *J. Clin. Neurosci.* **57**, 121–125. 10.1016/j.jocn.2018.08.015 (2018). 30143414 10.1016/j.jocn.2018.08.015 PMC6191314

[94] Sue, K., Hirabayashi, H., Osawa, M. & Komatsu, T. Relationship between neuropsychological test scores and hippocampal atrophy in non-demented Japanese older adults. *Interdiscip Neurosur*. **30**10.1016/j.inat.2022.101605 (2022).

[95] Frank, L. M., Brown, E. N. & Wilson, M. Trajectory encoding in the hippocampus and entorhinal cortex. *Neuron* **27**, 169–178. 10.1016/s0896-6273(00)00018-0 (2000). 10939340 10.1016/s0896-6273(00)00018-0

[96] Solstad, T., Boccara, C. N., Kropff, E. & Moser, M. B. Moser, E. I. Representation of geometric borders in the entorhinal cortex. *Science* **322**, 1865–1868. 10.1126/science.1166466 (2008). 19095945 10.1126/science.1166466

[97] Doeller, C. F., Barry, C. & Burgess, N. Evidence for grid cells in a human memory network. *Nature* **463**, 657–661. 10.1038/nature08704 (2010). 20090680 10.1038/nature08704 PMC3173857

[98] Rodrigue, K. M. & Raz, N. Shrinkage of the entorhinal cortex over five years predicts memory performance in healthy adults. *J. Neurosci.* **24**, 956–963. 10.1523/JNEUROSCI.4166-03.2004 (2004). 14749440 10.1523/JNEUROSCI.4166-03.2004 PMC6729806

[99] Newton, C. et al. Entorhinal-based path integration selectively predicts midlife risk of alzheimer’s disease. *Alzheimers Dement.* **20**, 2779–2793. 10.1002/alz.13733 (2024). 38421123 10.1002/alz.13733 PMC11032581

[100] Laczo, M. et al. Spatial navigation questionnaires as a supportive diagnostic tool in early alzheimer’s disease. *iScience* **27**, 109832. 10.1016/j.isci.2024.109832 (2024). 38779476 10.1016/j.isci.2024.109832 PMC11108981

[101] Hyde, J. S. The gender similarities hypothesis. *Am. Psychol.* **60**, 581–592. 10.1037/0003-066X.60.6.581 (2005). 16173891 10.1037/0003-066X.60.6.581

[102] Lawton, C. A. & Gender Spatial Abilities, and Wayfinding. *Gender Research in General and Experimental Psychology.* 1, 317–341 (2010). 10.1007/978-1-4419-1465-1_16

[103] Nazareth, A., Huang, X., Voyer, D. & Newcombe, N. A meta-analysis of sex differences in human navigation skills. *Psychon Bull. Rev.* **26**, 1503–1528. 10.3758/s13423-019-01633-6 (2019). 31270765 10.3758/s13423-019-01633-6

[104] Munion, A. K., Stefanucci, J. K., Rovira, E., Squire, P. & Hendricks, M. Gender differences in Spatial navigation: characterizing wayfinding behaviors. *Psychon Bull. Rev.* **26**, 1933–1940. 10.3758/s13423-019-01659-w (2019). 31432331 10.3758/s13423-019-01659-w

[105] Lawton, C. A. & Kallai, J. Gender differences in wayfinding strategies and anxiety about wayfinding: A Cross-Cultural comparison. *Sex. Roles*. **47**, 389–401. 10.1023/a:1021668724970 (2002).

[106] Boone, A. P., Gong, X. & Hegarty, M. Sex differences in navigation strategy and efficiency. *Mem. Cognit.* **46**, 909–922. 10.3758/s13421-018-0811-y (2018). 29790097 10.3758/s13421-018-0811-y

[107] Picucci, L., Caffò, A. O. & Bosco, A. Besides navigation accuracy: gender differences in strategy selection and level of Spatial confidence. *J. Environ. Psychol.* **31**, 430–438. 10.1016/j.jenvp.2011.01.005 (2011).

[108] Lawton, C. A. Gender differences in way-finding strategies: relationship to Spatial ability and Spatial anxiety. *Sex. Roles*. **30**, 765–779. 10.1007/bf01544230 (1994).

[109] Gavrielidou, E. & Lamers, M. H. Landmarks and Time-Pressure in Virtual Navigation: Towards Designing Gender-Neutral Virtual Environments. *International Conference on Facets of Virtual Environments* 60–67 (2010). 10.1007/978-3-642-11743-5_5

[110] Schinazi, V. R. et al. Motivation moderates gender differences in navigation performance. *Sci. Rep.* **13**, 15995. 10.1038/s41598-023-43241-4 (2023). 37749312 10.1038/s41598-023-43241-4 PMC10520045

[111] Ross, S. P., Skelton, R. W. & Mueller, S. C. Gender differences in Spatial navigation in virtual space: implications when using virtual environments in instruction and assessment. *Virtual Real.* **10**, 175–184. 10.1007/s10055-006-0041-7 (2006).

[112] Padilla, L. M., Creem-Regehr, S. H., Stefanucci, J. K. & Cashdan, E. A. Sex differences in virtual navigation influenced by scale and navigation experience. *Psychon. Bull. Rev.* **24**, 582–590. 10.3758/s13423-016-1118-2 (2017). 27714666 10.3758/s13423-016-1118-2

[113] Saucier, D., Bowman, M. & Elias, L. Sex differences in the effect of articulatory or Spatial dual-task interference during navigation. *Brain Cogn.* **53**, 346–350. 10.1016/s0278-2626(03)00140-4 (2003). 14607178 10.1016/s0278-2626(03)00140-4

[114] Coutrot, A. et al. Global determinants of navigation ability. *Curr. Biol.* **28**, 2861–2866e2864. 10.1016/j.cub.2018.06.009 (2018). 30100340 10.1016/j.cub.2018.06.009

[115] Global Gender Gap Report. : Insight Report. 395 (World Economic Forum, 2025). (2025).

[116] Broekhuis, M., van Velsen, L., Stal, S., Weldink, J. & Tabak, M. ter in *5th International Conference on Information and Communication Technologies for Ageing Well and e-Health, ICT4AWE 2019.* 48–57 (SCITEPRESS).

[117] Marston, H. R. Design recommendations for digital game design within an ageing society. *Educ. Gerontol.* **39**, 103–118. 10.1080/03601277.2012.689936 (2013). https://doi.org/

[118] Martinho, D., Carneiro, J., Corchado, J. M. & Marreiros, G. A systematic review of gamification techniques applied to elderly care. *Artif. Intell. Rev.* **53**, 4863–4901. 10.1007/s10462-020-09809-6 (2020).

[119] Ijsselsteijn, W., Nap, H. H., De Kort, Y. & Poels, K. in *Proceedings of the 2007 conference on Future Play.* 17–22.

[120] Czaja, S. J. Usability of technology for older adults: where are we and where do we need to be. *J. Usability Stud.* **14**, 61–64 (2019).

[121] Czaja, S. J., Boot, W. R., Charness, N. & Rogers, W. A. *Designing for Older Adults: Principles and Creative Human Factors Approaches* 3rd edn (CRC, 2019).

[122] Lorenz, M., Brade, J., Klimant, P., Heyde, C. E. & Hammer, N. Age and gender effects on presence, user experience and usability in virtual environments–first insights. *PLOS ONE*. **18**, e0283565. 10.1371/journal.pone.0283565 (2023). 36972245 10.1371/journal.pone.0283565 PMC10042342

[123] Sonderegger, A., Schmutz, S. & Sauer, J. The influence of age in usability testing. *Appl. Ergon.* **52**, 291–300. 10.1016/j.apergo.2015.06.012 (2016). 26360221 10.1016/j.apergo.2015.06.012

[124] Hartley, T. & Harlow, R. An association between human hippocampal volume and topographical memory in healthy young adults. *Front. Hum. Neurosci.* **6**10.3389/fnhum.2012.00338 (2012). 10.3389/fnhum.2012.00338 PMC3533499 23293595

[125] Farias, S. T. et al. The measurement of everyday cognition (ECog): scale development and psychometric properties. *Neuropsychology* **22**, 531–544. 10.1037/0894-4105.22.4.531 (2008). 18590364 10.1037/0894-4105.22.4.531 PMC2877034

[126] Hegarty, M., Richardson, A. E., Montello, D. R., Lovelace, K. & Subbiah, I. Development of a self-report measure of environmental Spatial ability. *Intelligence* **30**, 425–447 (2002).

[127] Lovibond, P. F. & Lovibond, S. H. The structure of negative emotional states: comparison of the depression anxiety stress scales (DASS) with the Beck depression and anxiety inventories. *Behav. Res. Ther.* **33**, 335–343. 10.1016/0005-7967(94)00075-u (1995). 7726811 10.1016/0005-7967(94)00075-u

[128] Mohs, R. C. et al. Development of cognitive instruments for use in clinical trials of antidementia drugs: additions to the alzheimer’s disease assessment scale that broaden its scope. The alzheimer’s disease cooperative study. *Alzheimer Dis. Assoc. Disord*. **11** (Suppl 2), S13–21 (1997). 9236948

[129] Reitan, R. M. W. *Deborah. The Halstead-Reitan Neuropsychological Test Battery: Theory and Clinical Interpretation* Vol. 4 (Neuropsychology, 1985).

[130] Della Sala, S., Foley, J. A., Beschin, N., Allerhand, M. & Logie, R. H. Assessing dual-task performance using a paper-and-pencil test: normative data. *Arch. Clin. Neuropsychol.* **25**, 410–419. 10.1093/arclin/acq039 (2010). 20513725 10.1093/arclin/acq039

[131] Foley, J. A., Kaschel, R., Logie, R. H. & Della Sala, S. Dual-task performance in alzheimer’s disease, mild cognitive impairment, and normal ageing. *Arch. Clin. Neuropsychol.* **26**, 340–348. 10.1093/arclin/acr032 (2011). 21576091 10.1093/arclin/acr032

[132] Friedman, A. & Kohler, B. Bidimensional regression: assessing the configural similarity and accuracy of cognitive maps and other Two-Dimensional data sets. *Psychol. Methods*. **8**, 468–491. 10.1037/1082-989x.8.4.468 (2003). 14664683 10.1037/1082-989X.8.4.468

[133] Tobler, W. R. Computation of the correspondence of geographical patterns. *Papers Reg. Sci. Association*. **15**, 131–139. 10.1007/bf01947869 (1965).

