# Supplementary material for: SPACE: A novel digital tool for assessing hippocampal structural integrity in older adults
Source: Sci Rep. 2026 Feb 12;16:8587. doi: 10.1038/s41598-026-39628-8 (PMC12976057; doi:10.1038/s41598-026-39628-8)
Supplement: Supplementary file 1 — Supplementary Material 1 [file 41598_2026_39628_MOESM1_ESM.docx]

## Supplementary Information 1

Using the same feature selection approach as for the hippocampus, we examined associations between entorhinal cortex volume with MoCA subdomains and standard neuropsychological tests (Supplementary Tables 1-2). We also applied this feature selection strategy to SPACE and to models including interaction terms between Path Integration (PI) and pointing error, and between PI and mapping performance (Supplementary Table 4). Across analyses, only a modest association between Trail Making Test A performance and right entorhinal cortex volume was observed.

**Supplementary Table 1.** Feature selection analysis of MoCA subdomains associated with entorhinal cortex volume.

|  | **Left EC** | | | | | | | | **Right EC** | | | |
| --- | --- | --- | --- | --- | --- | --- | --- | --- | --- | --- | --- | --- |
| **Predictor** | | **Est.** | | **SE** | **t** | **p** | **Est.** | **SE** | | **t** | **p** | |
|  | | | R^2^ = 0.39/ R^2^_adj_ = 0.20 | | | | R^2^ = 0.35/ R^2^_adj_ = 0.16 | | | | | |
| *Intercept* | | *394.860* | | *2032.213* | *0.194* | *0.847* | *2504.582* | *1743.513* | | *1.437* | | *0.161* |
| Age | | **-31.437** | | **11.949** | **-2.631** | **0.013** | **-22.353** | **10.252** | | **-2.180** | | **0.037** |
| Education | | 80.871 | | 187.226 | 0.432 | 0.669 | -12.308 | 160.629 | | -0.077 | | 0.939 |
| Visuospatial | | 149.646 | | 108.603 | 1.378 | 0.178 | 81.183 | 93.174 | | 0.871 | | 0.391 |
| Naming | | -11.351 | | 443.397 | -0.026 | 0.980 | 174.615 | 380.407 | | 0.459 | | 0.650 |
| Attention | | -62.105 | | 80.568 | -0.771 | 0.447 | -87.183 | 69.122 | | -1.261 | | 0.217 |
| Language | | 230.042 | | 146.407 | 1.571 | 0.127 | 169.820 | 125.608 | | 1.352 | | 0.186 |
| Abstraction | | -210.838 | | 125.625 | -1.678 | 0.104 | 44.969 | 107.779 | | 0.417 | | 0.679 |
| Delayed recall | | 15.270 | | 55.748 | 0.274 | 0.786 | 26.025 | 47.829 | | 0.544 | | 0.590 |
| Orientation | | 400.188 | | 238.990 | 1.675 | 0.104 | -100.657 | 205.038 | | -0.491 | | 0.627 |

Abbreviations: Est.: Estimate; SE: Standard Error. The variables with a significant (*p*<0.05) contribution to the model are marked in bold, and effect sizes are provided as Cohen’s f^2^. For the model fit measures, the number of stars designates the significance level: *** < 0.001, ** < 0.01, * < 0.05.

**Supplementary Table 2.** Feature selection analysis of standard neuropsychological test measures associated with entorhinal cortex volume.

|  | **Left EC** | | | | **Right EC** | | | |
| --- | --- | --- | --- | --- | --- | --- | --- | --- |
| **Predictor** | **Est.** | **SE** | **t** | **p** | **Est.** | **SE** | **t** | **p** |
|  | R^2^ = 0.23 / R^2^_adj_ = 0.06 | | | | R^2^ = 0.42 / R^2^_adj_ = 0.29* (f^2^= 0.72) | | | |
| *Intercept* | *2571.122* | *993.635* | *2.588* | *0.014* | *3245.889* | *717.108* | *4.526* | *<.001* |
| Age | -24.872 | 13.344 | -1.864 | 0.072 | -13.917 | 9.631 | -1.445 | 0.158 |
| Education | 111.310 | 184.285 | 0.604 | 0.550 | 90.035 | 132.999 | 0.677 | 0.503 |
| Maze Task | 0.838 | 7.982 | 0.105 | 0.917 | -2.786 | 5.761 | -0.484 | 0.632 |
| TMT-A | -2.128 | 4.775 | -0.446 | 0.659 | **-8.442** | **3.446** | **-2.450** | **0.020** |
| TMT-B | 0.683 | 1.066 | 0.641 | 0.526 | 0.379 | 0.769 | 0.492 | 0.626 |
| D-CAT | 9.689 | 12.618 | 0.768 | 0.448 | -9.390 | 9.107 | -1.031 | 0.310 |
| Dual-Task | 0.940 | 5.465 | 0.172 | 0.865 | -2.691 | 3.944 | -0.682 | 0.500 |

Abbreviations: Est.: Estimate; SE: Standard Error, TMT: Trail Making Test; D-CAT: Digit Cancellation Test. The variables with a significant (p<0.05) contribution to the model are marked in bold, and effect sizes are provided as Cohen’s f^2^. For the model fit measures, the number of stars designates the significance level: *** < 0.001, ** < 0.01, * < 0.05.

**Supplementary Table 3.** Demographic and SPACE variables as predictors of the entorhinal cortex volume.

|  | **Left EC** | | | | **Right EC** | | | |
| --- | --- | --- | --- | --- | --- | --- | --- | --- |
| **Predictor** | **Est.** | **SE** | **t** | **p** | **Est.** | **SE** | **t** | **p** |
|  | R^2^ = 0.29/R^2^_adj_= 0.13 | | | | R^2^ = 0.30/R^2^_adj_= 0.15 | | | |
| *Intercept* | *3424.701* | *960.296* | *3.566* | *0.001* | *3197.363* | *792.753* | *4.033* | *<0.001* |
| Age | -17.061 | 13.339 | -1.279 | 0.210 | -15.129 | 11.011 | -1.374 | 0.179 |
| Education | 27.840 | 167.529 | 0.166 | 0.869 | 7.015 | 138.300 | 0.051 | 0.960 |
| VS Training | -1.151 | 1.824 | -0.631 | 0.532 | -1.509 | 1.506 | -1.002 | 0.324 |
| PI | -0.321 | 0.602 | -0.534 | 0.597 | -0.332 | 0.497 | -0.668 | 0.509 |
| Pointing | -4.275 | 4.956 | -0.863 | 0.395 | -1.810 | 4.091 | -0.442 | 0.661 |
| Mapping | -158.790 | 215.396 | -0.737 | 0.466 | 2.457 | 177.815 | 0.014 | 0.989 |
| Perspective | -3.940 | 2.768 | -1.423 | 0.164 | -3.529 | 2.285 | -1.544 | 0.132 |
|  | R^2^ = 0.22/R^2^_adj_= 0.15* (f^2^ = 0.28) | | | | R^2^ = 0.21/R^2^_adj_= 0.15* (f^2^ = 0.27) | | | |
| *Intercept* | *2936.084* | *838.871* | *3.500* | *0.001* | *2678.930* | *701.104* | *3.821* | *<.001* |
| Age | -22.668 | 12.151 | -1.865 | 0.070 | -18.287 | 10.156 | -1.801 | 0.080 |
| Education | 128.381 | 143.571 | 0.894 | 0.377 | 128.172 | 119.992 | 1.068 | 0.293 |
| PI x Pointing | -0.008 | 0.009 | -0.937 | 0.355 | -0.005 | 0.007 | -0.700 | 0.488 |
|  | R^2^ = 0.23/R^2^_adj_= 0.17* (f^2^ = 0.30) | | | | R^2^ = 0.21/R^2^_adj_= 0.14* (f^2^ = 0.26) | | | |
| *Intercept* | *3003.949* | *832.133* | *3.610* | *<.001* | *2710.969* | *702.726* | *3.858* | *<.001* |
| Age | **-24.042** | **11.814** | **-2.035** | **0.049** | -19.328 | 9.977 | -1.937 | 0.061 |
| Education | 144.063 | 142.352 | 1.012 | 0.318 | 136.024 | 120.215 | 1.132 | 0.265 |
| PI x Mapping | -1.196 | 0.957 | -1.249 | 0.220 | -0.513 | 0.808 | -0.635 | 0.529 |

Abbreviations: EC: Entorhinal Cortex; Est.: Estimate; SE: Standard Error; VS: Visuospatial; PI: Path Integration. The variables with a significant (*p*<0.05) contribution to the model are marked in bold, and effect sizes are provided as Cohen’s f^2^. For the model fit measures, the number of stars designates the significance level: *** < 0.001, ** < 0.01, * < 0.05.

**Supplementary Information 2**

To examine whether performance on the SPACE tasks was associated with response-based navigation, we fit regression models predicting left and right caudate volumes while controlling for age and education. Models included individual SPACE task measures as well as interaction terms between PI and pointing error and between PI and mapping performance. No SPACE measures or interaction terms were associated with caudate volume.

**Supplementary Table 4.** Hierarchical regression models of caudate volume with SPACE tasks and interaction terms.

|  | **Left Caudate** | | | | **Right Caudate** | | | |
| --- | --- | --- | --- | --- | --- | --- | --- | --- |
| **Predictor** | **Est.** | **SE** | **t** | **p** | **Est.** | **SE** | **t** | **p** |
|  | R^2^ = 0.01/R^2^_adj_= –0.04* (f^2^ = 0.01) | | | | R^2^ = 0.03/R^2^_adj_= –0.02 | | | |
| *Intercept* | *2059.510* | *942.226* | *2.186* | *0.035* | *1677.850* | *996.867* | *1.683* | *0.101* |
| Age | 6.679 | 13.367 | 0.500 | 0.620 | 14.853 | 14.142 | 1.050 | 0.300 |
| Education | 83.129 | 161.143 | 0.516 | 0.609 | 54.533 | 170.487 | 0.320 | 0.751 |
|  | R^2^ = 0.08/R^2^_adj_= –0.12 | | | | R^2^ = 0.13/R^2^_adj_= –0.06 | | | |
| *Intercept* | *2505.707* | *1102.483* | *2.273* | *0.030* | *2,309* | *1,148* | *2.012* | *0.053* |
| Age | 1.572 | 15.314 | 0.103 | 0.919 | 11.43 | 15.94 | 0.717 | 0.478 |
| Education | 28.876 | 192.335 | 0.150 | 0.882 | -0.484 | 200.2 | -0.002 | 0.998 |
| VS Training | -1.641 | 2.094 | -0.783 | 0.439 | -2.424 | 2.180 | -1.112 | 0.274 |
| PI | -0.305 | 0.691 | -0.441 | 0.662 | -0.077 | 0.719 | -0.107 | 0.915 |
| Pointing | 5.835 | 5.689 | 1.026 | 0.313 | 5.292 | 5.922 | 0.894 | 0.378 |
| Mapping | -75.362 | 247.288 | -0.305 | 0.763 | -274.3 | 257.4 | -1.066 | 0.295 |
| Perspective | -0.103 | 3.178 | -0.033 | 0.974 | -0.063 | 3.309 | -0.019 | 0.985 |
|  | R^2^ = 0.01/R^2^_adj_= –0.04 | | | | R^2^ = 0.03 /R^2^_adj_= –0.02 | | | |
| *Intercept* | *2058.449* | *955.348* | *2.155* | *0.038* | *1680.692* | *1010.465* | *1.663* | *0.105* |
| Age | 6.844 | 13.839 | 0.495 | 0.624 | 14.395 | 14.637 | 0.983 | 0.332 |
| Education | 82.720 | 163.506 | 0.506 | 0.616 | 55.668 | 172.939 | 0.322 | 0.749 |
| PI x Pointing | –0.001 | 0.010 | –0.059 | 0.953 | 0.002 | 0.011 | 0.154 | 0.878 |
|  | R^2^ = 0.03/R^2^_adj_= –0.05 | | | | R^2^ = 0.11/R^2^_adj_=0.03 | | | |
| *Intercept* | *2105.814* | *944.417* | *2.230* | *0.032* | *1764.704* | *971.294* | *1.817* | *0.078* |
| Age | 7.495 | 13.408 | 0.559 | 0.580 | 16.383 | 13.789 | 1.188 | 0.243 |
| Education | 91.824 | 161.560 | 0.568 | 0.573 | 70.844 | 166.158 | 0.426 | 0.672 |
| PI x Mapping | -1.044 | 1.086 | -0.962 | 0.343 | -1.959 | 1.117 | -1.754 | 0.088 |

Abbreviations: Est.: Estimate; SE: Standard Error; VS: Visuospatial; PI: Path Integration. The variables with a significant (*p*<0.05) contribution to the model are marked in bold, and effect sizes are provided as Cohen’s f^2^. For the model fit measures, the number of stars designates the significance level: *** < 0.001, ** < 0.01, * < 0.05.

## Supplementary Information 3

To examine the robustness of the association between navigation performance and hippocampal volume, we reduced model complexity and conducted a set of domain-specific hierarchical regression analyses using the MoCA and the neuropsychological test battery. Given the modest sample size (n = 40), predictors were grouped by cognitive domain to limit the number of variables entered simultaneously and to mitigate potential overfitting. Three theory-driven models were estimated: a global cognition model, a visuospatial model, and an attention model. In each model, age and education were entered in the first block, followed by domain-specific neuropsychological measures in the second block, and the PI × Mapping interaction term in the final block. Across all three models, the PI × Mapping interaction term consistently emerged as a significant predictor of left and right hippocampal volume, whereas none of the neuropsychological measures were significant (see Supplementary Tables 5–7).

**Supplementary Table 5.** Global cognition model.

|  | **Left hippocampus** | | | | **Right hippocampus** | | | |
| --- | --- | --- | --- | --- | --- | --- | --- | --- |
| **Predictor** | **Est.** | **SE** | **t** | **p** | **Est.** | **SE** | **t** | **p** |
| **Model 1** | R^2^ = 0.20/R^2^_adj_=0.15* (f^2^ = 0.25) | | | | R^2^ = 0.37/R^2^_adj_=0.34*** (f^2^ = 0.59) | | | |
| *Intercept* | *3415.461* | *598.445* | *5.707* | *<0.001* | *3776.494* | *585.628* | *6.449* | *<0.001* |
| Age | -13.598 | 8.490 | -1.602 | 0.118 | **-19.132** | **8.308** | **-2.303** | **0.027** |
| Education | 155.619 | 102.348 | 1.520 | 0.137 | **252.665** | **100.156** | **2.523** | **0.016** |
| **Model 2** | R^2^ = 0.22/R^2^_adj_=0.16* (f^2^ = 0.28) | | | | R^2^ = 0.39/R^2^_adj_=0.34*** (f^2^ = 0.64) | | | |
| *Intercept* | *2781.129* | *828.038* | *3.359* | *0.002* | *3119.325* | *808.643* | *3.857* | *<0.001* |
| Age | -12.455 | 8.528 | -1.461 | 0.153 | **-17.948** | **8.328** | **-2.155** | **0.038** |
| Education | 67.216 | 129.673 | 0.518 | 0.607 | 161.079 | 126.636 | 1.272 | 0.212 |
| MoCA | 22.978 | 20.797 | 1.105 | 0.277 | 23.805 | 20.310 | 1.172 | 0.249 |
| **Model 3** | R^2^ = 0.47/R^2^_adj_=0.41*** (f^2^ = 0.89) | | | | R^2^ = 0.51/R^2^_adj_=0.46***(f^2^ = 1.04) | | | |
| *Intercept* | *3488.692* | *718.694* | *4.854* | *<0.001* | *3665.569* | *759.275* | *4.828* | *<0.001* |
| Age | -11.653 | 7.175 | -1.624 | 0.113 | **-17.328** | **7.580** | **-2.286** | **0.028** |
| Education | 171.006 | 112.128 | 1.525 | 0.136 | **241.206** | **118.459** | **2.036** | **0.049** |
| MoCA | 1.223 | 18.323 | 0.067 | 0.947 | 7.010 | 19.358 | 0.362 | 0.719 |
| PI x Mapping | **-2.413** | **0.605** | **-3.986** | **<0.001** | **-1.863** | **0.640** | **-2.913** | **0.006** |
| **Model comparison** | | | | | | | | |
|  | **Left hippocampus** | | | | **Right hippocampus** | | | |
| **Models** | **∆R^2^** | **F** | **df1/df2** | **p** | **∆R^2^** | **F** | **df1/df2** | **p** |
| 1-2 | 0.03 | 1.221 | 1/36 | 0.277 | 0.02 | 1.374 | 1/36 | 0.249 |
| 2-3 | **0.24** | **15.892** | **1/35** | **<0.001** | **0.12** | **8.486** | **1/35** | **0.006** |

Abbreviations: Est.: Estimate; SE: Standard Error; MoCA: Montreal Cognitive Assessment; PI: Path Integration. The variables with a significant (*p*<0.05) contribution to the model are marked in bold, and effect sizes are provided as Cohen’s f^2^. For the model fit measures, the number of stars designates the significance level: *** < 0.001, ** < 0.01, * < 0.05.

**Supplementary Table 6.** Visuospatial model.

|  | **Left hippocampus** | | | | **Right hippocampus** | | | |
| --- | --- | --- | --- | --- | --- | --- | --- | --- |
| **Predictor** | **Est.** | **SE** | **t** | **p** | **Est.** | **SE** | **t** | **p** |
| **Model 1** | R^2^ = 0.20/R^2^_adj_=0.15* (f^2^ = 0.25) | | | | R^2^ = 0.37/R^2^_adj_=0.34*** (f^2^ = 0.59) | | | |
| *Intercept* | *3415.461* | *598.445* | *5.707* | *<0.001* | *3776.494* | *585.628* | *6.449* | *<0.001* |
| Age | -13.598 | 8.490 | -1.602 | 0.118 | **-19.132** | **8.308** | **-2.303** | **0.027** |
| Education | 155.619 | 102.348 | 1.520 | 0.137 | **252.665** | **100.156** | **2.523** | **0.016** |
| **Model 2** | R^2^ = 0.26/R^2^_adj_=0.15 | | | | R^2^ = 0.38/R^2^_adj_=0.29** (f^2^ = 0.61) | | | |
| *Intercept* | *3478.856* | *603.761* | *5.762* | *<0.001* | *3834.231* | *608.835* | *6.298* | *<0.001* |
| Age | -13.212 | 8.615 | -1.533 | 0.134 | **-17.947** | **8.688** | **-2.066** | **0.047** |
| Education | 127.600 | 113.702 | 1.122 | 0.270 | 220.753 | 114.658 | 1.925 | 0.063 |
| Maze task | -7.016 | 5.397 | -1.300 | 0.202 | -4.438 | 5.443 | -0.815 | 0.420 |
| TMT-A | 4.707 | 3.029 | 1.554 | 0.129 | 0.364 | 3.054 | 0.119 | 0.906 |
| TMT-B | -0.576 | 0.712 | -0.808 | 0.425 | -0.053 | 0.718 | -0.074 | 0.942 |
| **Model 3** | R^2^ = 0.53/R^2^_adj_=0.44*** (f^2^ = 1.13) | | | | R^2^ = 0.54/R^2^_adj_=0.46***(f^2^ =1.17) | | | |
| *Intercept* | *3574.921* | *489.698* | *7.300* | *<0.001* | *3914.881* | *535.218* | *7.315* | *<0.001* |
| Age | -10.683 | 7.005 | -1.525 | 0.137 | **-15.825** | **7.656** | **-2.067** | **0.047** |
| Education | 162.062 | 92.469 | 1.753 | 0.089 | **249.685** | **101.065** | **2.471** | **0.019** |
| Maze task | -8.477 | 4.386 | -1.933 | 0.062 | -5.665 | 4.794 | -1.182 | 0.246 |
| TMT-A | 3.296 | 2.475 | 1.332 | 0.192 | -0.819 | 2.706 | -0.303 | 0.764 |
| TMT-B | -0.012 | 0.591 | -0.021 | 0.984 | 0.420 | 0.646 | 0.650 | 0.520 |
| PI x Mapping | **-2.493** | **0.575** | **-4.335** | **<0.001** | **-2.093** | **0.629** | **-3.330** | **0.002** |
| **Model comparison** | | | | | | | | |
|  | **Left hippocampus** | | | | **Right hippocampus** | | | |
| **Models** | **∆R^2^** | **F** | **df1/df2** | **p** | **∆R^2^** | **F** | **df1/df2** | **p** |
| 1-2 | 0.06 | 0.983 | 3/34 | 0.412 | 0.01 | 0.265 | 3/34 | 0.850 |
| 2-3 | **0.27** | **18.790** | **1/33** | **<0.001** | **0.15** | **11.087** | **1/33** | **0.002** |

Abbreviations: Est.: Estimate; SE: Standard Error; TMT: Trail Making Test; PI: Path Integration distance error. The variables with a significant (*p*<0.05) contribution to the model are marked in bold, and effect sizes are provided as Cohen’s f^2^. For the model fit measures, the number of stars designates the significance level: *** < 0.001, ** < 0.01, * < 0.05.

**Supplementary Table 7.** Attention model.

|  | **Left hippocampus** | | | | **Right hippocampus** | | | |
| --- | --- | --- | --- | --- | --- | --- | --- | --- |
| **Predictor** | **Est.** | **SE** | **t** | **p** | **Est.** | **SE** | **t** | **p** |
| **Model 1** | R^2^ = 0.20/R^2^_adj_ = 0.15* (f^2^ = 0.25) | | | | R^2^ = 0.37/R^2^_adj_=0.34*** (f^2^ = 0.59) | | | |
| *Intercept* | *3415.461* | *598.445* | *5.707* | *<0.001* | *3776.494* | *585.628* | *6.449* | *<0.001* |
| Age | -13.598 | 8.490 | -1.602 | 0.118 | **-19.132** | **8.308** | **-2.303** | **0.027** |
| Education | 155.619 | 102.348 | 1.520 | 0.137 | **252.665** | **100.156** | **2.523** | **0.016** |
| **Model 2** | R^2^ = 0.20/R^2^_adj_ = 0.11 | | | | R^2^ = 0.39/R^2^_adj_=0.32** (f^2^ = 0.64) | | | |
| *Intercept* | *3416.020* | *662.336* | *5.158* | *<0.001* | *3537.418* | *635.962* | *5.562* | *<0.001* |
| Age | -13.980 | 9.212 | -1.518 | 0.138 | **-18.363** | **8.845** | **-2.076** | **0.045** |
| Education | 151.713 | 118.994 | 1.275 | 0.211 | 223.490 | 114.256 | 1.956 | 0.058 |
| D-CAT | -0.578 | 7.192 | -0.080 | 0.936 | 8.059 | 6.905 | 1.167 | 0.251 |
| Dual Task | 0.444 | 3.688 | 0.120 | 0.905 | -0.251 | 3.541 | -0.071 | 0.944 |
| **Model 3** | R^2^ = 0.48/R^2^_adj_ = 0.41*** (f^2^ = 0.92) | | | | R^2^ = 0.54/R^2^_adj_=0.47***(f^2^ = 1.17) | | | |
| *Intercept* | *3448.498* | *538.968* | *6.398* | *<0.001* | *3562.885* | *563.300* | *6.325* | *<0.001* |
| Age | -14.267 | 7.496 | -1.903 | 0.065 | **-18.589** | **7.834** | **-2.373** | **0.023** |
| Education | 136.450 | 96.885 | 1.408 | 0.168 | **211.521** | **101.259** | **2.089** | **0.044** |
| D-CAT | -1.596 | 5.856 | -0.272 | 0.787 | 7.261 | 6.121 | 1.186 | 0.244 |
| Dual Task | 3.336 | 3.074 | 1.085 | 0.285 | 2.017 | 3.213 | 0.628 | 0.534 |
| PI x Mapping | **-2.565** | **0.590** | **-4.344** | **<.001** | **-2.011** | **0.617** | **-3.259** | **0.003** |
| **Model comparison** | | | | | | | | |
|  | **Left hippocampus** | | | | **Right hippocampus** | | | |
| **Models** | **∆R^2^** | **F** | **df1/df2** | **p** | **∆R^2^** | **F** | **df1/df2** | **p** |
| 1-2 | 0.00 | 0.009 | 2/35 | 0.991 | 0.02 | 0.687 | 2/35 | 0.510 |
| 2-3 | **0.29** | **18.867** | **1/34** | **<0.001** | **0.14** | **10.621** | **1/34** | **0.003** |

Abbreviations: Est.: Estimate; SE: Standard Error; D-CAT: Digit Cancellation Test; PI: Path Integration distance error. The variables with a significant (*p*<0.05) contribution to the model are marked in bold, and effect sizes are provided as Cohen’s f^2^. For the model fit measures, the number of stars designates the significance level: *** < 0.001, ** < 0.01, * < 0.05.

## Supplementary Information 4

We conducted linear regression analyses to assess whether visual impairments or prior tablet experience influenced performance on the SPACE navigation tasks while controlling for age and education. Visual defects were not significantly associated (all p ≥ 0.30) with performance in visuospatial training, PI, pointing, or perspective taking (Supplementary Table 8). A small association was observed between mapping performance and visual defect reporting (*β* = 0.255, p = 0.020), suggesting slightly higher mapping accuracy among participants who reported visual defects. However, this effect did not survive correction for multiple comparisons (α = 0.013). Tablet experience was also not a significant predictor (all p ≥ 0.169) in any of these models (Supplementary Table 9).

**Supplementary Table 8.** The effect of visual defects on SPACE performance.

| **VS training time** | | | | |
| --- | --- | --- | --- | --- |
| Predictor | **Est.** | **SE** | **t** | **p** |
| Model | R^2^ = 0.22/R^2^_adj_= 0.15* (f^2^ = 0.28) | | | |
| *Intercept* | *237.0* | *79.07* | *2.997* | *0.005* |
| Age | 0.585 | 1.120 | 0.523 | 0.604 |
| Education | **-32.95** | **13.43** | **-2.453** | **0.019** |
| Visual defects | 12.43 | 13.11 | 0.948 | 0.349 |
| **PI distance error** | | | | |
| Predictor | **Est.** | **SE** | **t** | **p** |
| Model | R^2^ = 0.01/R^2^_adj_=-0.07 | | | |
| *Intercept* | *221.4* | *260.3* | *0.851* | *0.401* |
| Age | 0.130 | 3.680 | 0.035 | 0.972 |
| Education | -25.75 | 44.22 | -0.582 | 0.564 |
| Visual defects | -4.437 | 43.16 | -0.103 | 0.919 |
| **Pointing error** | | | | |
| Predictor | **Est.** | **SE** | **t** | **p** |
| Model | R^2^ = 0.20/R^2^_adj_=0.13* (f^2^ = 0.25) | | | |
| *Intercept* | *4.570* | *30.24* | *0.151* | *0.881* |
| Age | **1.104** | **0.427** | **2.584** | **0.014** |
| Education | -0.431 | 5.138 | -0.084 | 0.934 |
| Visual defects | 3.216 | 5.015 | 0.641 | 0.525 |
| **Mapping performance** | | | | |
| Predictor | **Est.** | **SE** | **t** | **p** |
| Model | R^2^ = 0.16/R^2^_adj_ = 0.16 | | | |
| *Intercept* | *0.020* | *0.635* | *0.031* | *0.975* |
| Age | 0.005 | 0.009 | 0.604 | 0.550 |
| Education | 0.092 | 0.108 | 0.851 | 0.400 |
| **Visual defects** | **0.255** | **0.105** | **2.425** | **0.020** |
| **Perspective error** | | | | |
| Predictor | **Est.** | **SE** | **t** | **p** |
| Model | R^2^ = 0.19/R^2^_adj_ = 0.12 | | | |
| *Intercept* | *11.35* | *52.09* | *0.218* | *0.829* |
| Age | 0.555 | 0.736 | 0.755 | 0.455 |
| Education | **-18.63** | **8.850** | **-2.105** | **0.042** |
| Visual defects | 5.830 | 8.638 | 0.675 | 0.504 |

Abbreviations: Est.: Estimate; SE: Standard Error. For the model fit measures, the number of stars designates the significance level: *** < 0.001, ** < 0.01, * < 0.05.

**Supplementary Table 9.** The effect of tablet experience on SPACE performance.

| **VS training time** | | | | |
| --- | --- | --- | --- | --- |
| Predictor | **Est.** | **SE** | **t** | **p** |
| Model | R^2^ = 0.23/R^2^_adj_ = 0.14 | | | |
| *Intercept* | *245.6* | *79.55* | *3.157* | *0.003* |
| Age | 0.453 | 1.130 | 0.401 | 0.691 |
| **Education** | **-32.16** | **14.27** | **-2.254** | **0.031** |
| Tablet experience: Low-None  High-None | 9.822  -9.743 | 19.24  13.23 | 0.511  -0.737 | 0.613  0.466 |
| **PI distance error** | | | | |
| Predictor | **Est.** | **SE** | **t** | **p** |
| Model | R^2^ = 0.07 /R^2^_adj_ = -0.03 | | | |
| *Intercept* | *197.5* | *255.2* | *0.774* | *0.444* |
| Age | 0.674 | 3.630 | 0.186 | 0.854 |
| Education | -5.668 | 45.76 | -0.124 | 0.902 |
| Tablet experience: Low-None  High-None | -86.68  -39.35 | 61.71  42.42 | -1.405  -0.928 | 0.169  0.360 |
| **Pointing error** | | | | |
| Predictor | **Est.** | **SE** | **t** | **p** |
| Model | R^2^ = 0.22/R^2^_adj_ = 0.13 | | | |
| *Intercept* | *6.320* | *30.15* | *0.210* | *0.835* |
| **Age** | **1.070** | **0.429** | **2.491** | **0.018** |
| Education | -1.980 | 5.407 | -0.366 | 0.717 |
| Tablet experience: Low-None  High-None | 2.450  5.850 | 7.291  5.013 | 0.336  1.168 | 0.739  0.251 |
| **Mapping performance** | | | | |
| Predictor | **Est.** | **SE** | **t** | **p** |
| Model | R^2^ = 0.04/R^2^_adj_ = -0.08 | | | |
| *Intercept* | *0.232* | *0.686* | *0.338* | *0.737* |
| Age | 0.003 | 0.010 | 0.354 | 0.726 |
| Education | 0.097 | 0.123 | 0.788 | 0.436 |
| Tablet experience: Low-None  High-None | 0.065  -0.059 | 0.166  0.114 | 0.392  -0.516 | 0.698  0.609 |
| **Perspective error** | | | | |
| Predictor | **Est.** | **SE** | **t** | **p** |
| Model | R^2^ = 0.22/R^2^_adj_ = 0.13 | | | |
| *Intercept* | *10.33* | *51.62* | *0.200* | *0.842* |
| Age | 0.619 | 0.734 | 0.843 | 0.405 |
| Education | -15.53 | 9.257 | -1.678 | 0.102 |
| Tablet experience: Low-None  High-None | -17.04  -3.481 | 12.48  8.581 | -1.365  -0.406 | 0.181  0.687 |

Abbreviations: Est.: Estimate; SE: Standard Error. For the model fit measures, the number of stars designates the significance level: *** < 0.001, ** < 0.01, * < 0.05.

**Supplementary Information 5**

This Supplementary section provides additional analyses to clarify the rationale for modelling the interaction term between PI distance error and mapping performance as a composite index of joint spatial efficiency, rather than as a conventional moderation model.

The primary aim of our analysis was to examine whether jointly efficient spatial encoding and reconstruction constitute a meaningful marker of hippocampal integrity rather than to test whether the effect of one spatial task depends on the level of another (i.e., moderation). PI and mapping rely on complementary hippocampal-dependent processes, including the accurate encoding of landmark positions during navigation and the reconstruction of these spatial relations during mapping. We therefore modelled the multiplicative term (PI distance error × mapping performance) as a single composite predictor indexing joint spatial efficiency. This approach is consistent with regression theory, which does not require main effects to be included when the theoretical interest lies in a specific composite term rather than conditional effects (Cleves et al., 2008).

To further clarify why mapping behaves inconsistently when entered as a main effect and to illustrate the logic of the synergy model, we conducted two additional analyses. First, we fitted regression models predicting left and right hippocampal volumes (controlling for age and education) and plotted the resulting residuals against mapping performance, with points colour-coded by PI error group (Supplementary Figure 1). If mapping were a clean hippocampal marker, higher mapping performance would be expected to align with larger residual hippocampal volumes, and individuals with good PI would cluster at high mapping values. Instead, both figures show a criss-crossing pattern in which some participants with poor PI achieved high mapping scores, while others with good PI produced poor maps. These patterns reinforce the idea that mapping performance reflects a combination of hippocampal-dependent and independent processes. Consequently, mapping alone misclassifies participants’ hippocampal-dependent spatial learning ability, explaining why its coefficient reverses when entered as a standalone predictor.

Second, to visualise how hippocampal volume relates to the joint efficiency of encoding and reconstruction, we computed the synergy score (PI error × mapping performance) and divided participants into quartiles of this distribution (Supplementary Figure 2). Because PI is an error measure (lower values indicate better performance) and mapping is a performance measure (higher values indicate better performance), multiplying the two produces a composite in which joint efficiency (low PI error combined with high mapping accuracy) yields smaller values, whereas joint inefficiency (high PI error combined with low mapping accuracy) yields larger values. Lower synergy values, therefore, reflect better joint spatial performance, whereas higher values indicate poorer combined performance. Participants in the best synergy quartile (Q1: low PI error and high mapping accuracy) showed the largest hippocampal volumes, while those in the worst quartile (Q4: high PI error and low mapping accuracy) showed the smallest volumes, with intermediate quartiles falling between these extremes.


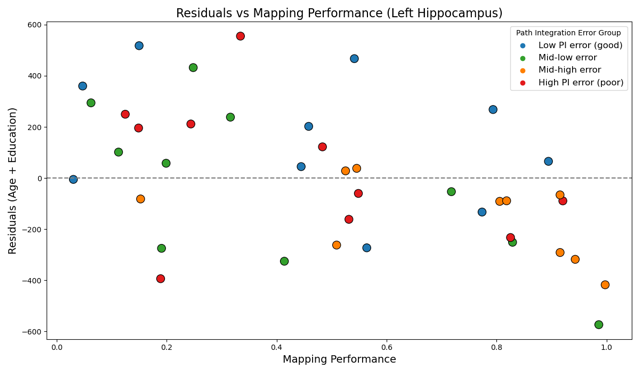


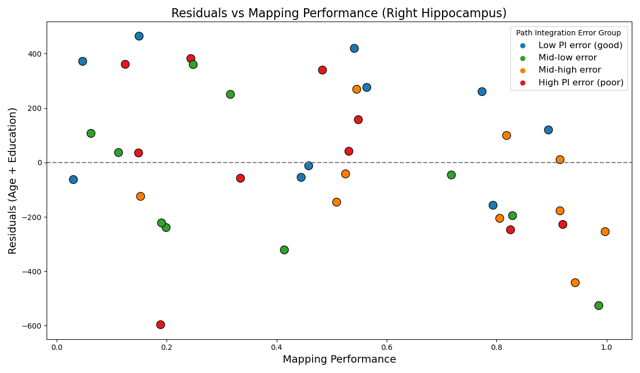


**Supplementary Figure 1.** Residual analysis of mapping performance for the left (top) and right (bottom) hippocampus. Points are colour-coded by PI performance (low vs high PI error). If mapping performance were a clean hippocampal marker, higher mapping scores would align with larger residual hippocampal volumes and cluster among participants with good PI performance. Instead, the criss-crossing pattern indicates that mapping performance reflects a mixture of hippocampal-dependent and hippocampal-independent processes.


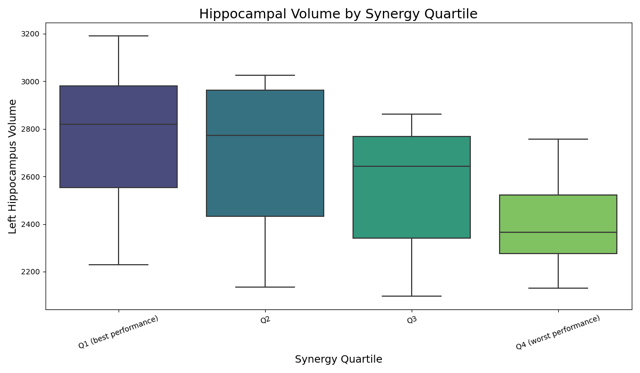


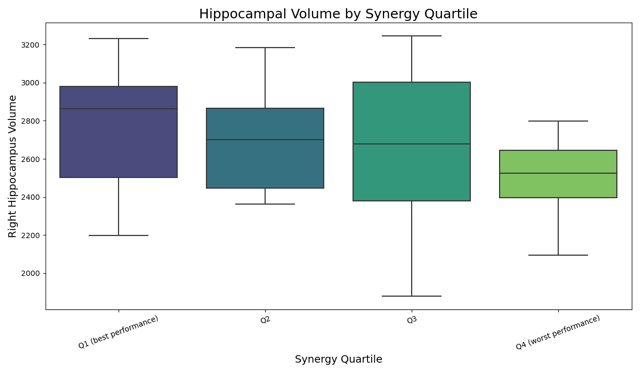


**Supplementary Figure 2.** Synergy quartiles of the interaction term (PI x mapping) for the right (top) and left (bottom) hippocampus. Participants were divided into quartiles based on the composite score. Lower values indicate better joint spatial efficiency (low PI distance error combined with high mapping accuracy). For both left (top) and right (bottom) hippocampus, participants in the best joint-efficiency quartile (Q1) showed the largest hippocampal volumes, whereas those in the poorest quartile (Q4) showed the smallest volumes. Intermediate quartiles fell between these extremes.

##

##

##

Supplementary Tables 10–12 present a series of regression models designed to clarify the analytical rationale underlying the composite PI × mapping predictor. Table 10 reports models in which age and education were entered in Step 1, followed by PI distance error and mapping performance entered as separate main effects in Step 2. In this formulation, mapping performance exhibits an unexpected negative coefficient, indicating that mapping accuracy, when dissociated from encoding performance, is an unstable marker of hippocampal volume. Table 11 presents models in which PI distance error, mapping performance, and their interaction term were entered simultaneously as a conventional moderation model. Under this specification, none of the coefficients reached statistical significance, indicating that moderation models test conditional effects rather than the joint efficiency of spatial encoding and reconstruction. Finally, Table 12 reports models in which the PI × mapping product term was entered as a single composite predictor alongside age and education. This formulation directly indexes joint spatial efficiency and yields a theoretically coherent association with hippocampal volume in both hemispheres.

**Supplementary Table 10.** Models with the main effects of PI distance error and mapping performance as independent contributors to hippocampus volume.

|  | **Left hippocampus** | | | | **Right hippocampus** | | | |
| --- | --- | --- | --- | --- | --- | --- | --- | --- |
| **Predictor** | **Est.** | **SE** | **t** | **p** | **Est.** | **SE** | **t** | **p** |
| **Model 1** | R^2^ = 0.20/R^2^_adj_=0.15* (f^2^ = 0.25) | | | | R^2^ = 0.37/R^2^_adj_=0.34*** (f^2^ = 0.59) | | | |
| *Intercept* | *3415.461* | *598.445* | *5.707* | *<.001* | *3776.494* | *585.628* | *6.449* | *<0.001* |
| Age | -13.598 | 8.490 | -1.602 | 0.118 | **-19.132** | **8.308** | **-2.303** | **0.027** |
| Education | 155.619 | 102.348 | 1.520 | 0.137 | **252.665** | **100.156** | **2.523** | **0.016** |
| **Model 2** | R^2^ =0.48/R^2^_adj_=0.43 (f^2^ =0.92) | | | | R^2^ = 0.54/R^2^_adj_=0.48*** (f^2^ = 1.17) | | | |
| *Intercept* | *3666.915* | *499.437* | *7.342* | *<0.001* | *4035.755* | *523.966* | *7.702* | *<0.001* |
| Age | -11.537 | 7.014 | -1.645 | 0.109 | **-17.593** | **7.358** | **-2.391** | **0.022** |
| Education | **185.798** | **85.338** | **2.177** | **0.036** | **265.078** | **89.530** | **2.961** | **0.005** |
| PI | **-0.690** | **0.323** | **-2.137** | **0.040** | **-0.856** | **0.339** | **-2.525** | **0.016** |
| Mapping | **-512.870** | **122.717** | **-4.179** | **<0.001** | **-368.574** | **128.744** | **-2.863** | **0.007** |
| **Model comparison** | | | | | | | | |
|  | **Left hippocampus** | | | | **Right hippocampus** | | | |
| **Models** | **∆R^2^** | **F** | **df1/df2** | **p** | **∆R^2^** | **F** | **df1/df2** | **p** |
| 1-2 | **0.29** | **9.734** | **2/35** | **<0.001** | **0.17** | **6.196** | **2/35** | **0.005** |

Abbreviations: Est.: Estimate; SE: Standard Error; PI: Path Integration. The variables with a significant (*p*<0.05) contribution to the model are marked in bold, and effect sizes are provided as Cohen’s f^2^. For the model fit measures, the number of stars designates the significance level: *** < 0.001, ** < 0.01, * < 0.05.

**Supplementary Table 11.** Models with the main effects and interaction of PI distance error and mapping performance as contributors to hippocampus volume.

|  | **Left hippocampus** | | | | **Right hippocampus** | | | |
| --- | --- | --- | --- | --- | --- | --- | --- | --- |
| **Predictor** | **Est.** | **SE** | **t** | **p** | **Est.** | **SE** | **t** | **p** |
| **Model 1** | R^2^ = 0.20/R^2^_adj_=0.15* (f^2^ = 0.25) | | | | R^2^ = 0.37/R^2^_adj_=0.34*** (f^2^ = 0.59) | | | |
| *Intercept* | *3415.461* | *598.445* | *5.707* | *<.001* | *3776.494* | *585.628* | *6.449* | *<0.001* |
| Age | -13.598 | 8.490 | -1.602 | 0.118 | **-19.132** | **8.308** | **-2.303** | **0.027** |
| Education | 155.619 | 102.348 | 1.520 | 0.137 | **252.665** | **100.156** | **2.523** | **0.016** |
| **Model 2** | R^2^ =0.49/R^2^_adj_=0.47*** (f^2^ =0.96) | | | | R^2^ = 0.54/R^2^_adj_=0.47*** (f^2^ =1.17) | | | |
| *Intercept* | *3650.199* | *514.457* | *7.095* | *<0.001* | *4004.832* | *539.149* | *7.428* | *<0.001* |
| Age | -11.544 | 7.113 | -1.623 | 0.114 | **-17.606** | **7.454** | **-2.362** | **0.024** |
| Education | **184.112** | **87.018** | **2.116** | **0.042** | **261.960** | **91.195** | **2.873** | **0.007** |
| PI | -0.605 | 0.564 | -1.073 | 0.291 | -0.699 | 0.591 | -1.182 | 0.246 |
| Mapping | -438.277 | 421.639 | -1.039 | 0.306 | -230.585 | 441.876 | -0.522 | 0.605 |
| PI x Mapping | -0.372 | 2.011 | -0.185 | 0.854 | -0.689 | 2.107 | -0.327 | 0.746 |
| **Model comparison** | | | | | | | | |
|  | **Left hippocampus** | | | | **Right hippocampus** | | | |
| **Models** | **∆R^2^** | **F** | **df1/df2** | **p** | **∆R^2^** | **F** | **df1/df2** | **p** |
| **1-2** | **0.29** | **6.322** | **3/34** | **0.002** | **0.17** | **4.061** | **3/34** | **0.014** |

Abbreviations: Est.: Estimate; SE: Standard Error; PI: Path Integration. The variables with a significant (*p*<0.05) contribution to the model are marked in bold, and effect sizes are provided as Cohen’s f^2^. For the model fit measures, the number of stars designates the significance level: *** < 0.001, ** < 0.01, * < 0.05.

**Supplementary Table 12.** The interaction of PI distance error and mapping performance as a contributor to hippocampus volume.

|  | **Left hippocampus** | | | | **Right hippocampus** | | | |
| --- | --- | --- | --- | --- | --- | --- | --- | --- |
| **Predictor** | **Est.** | **SE** | **t** | **p** | **Est.** | **SE** | **t** | **p** |
| **Model 1** | R^2^ = 0.20/R^2^_adj_=0.15* (f^2^ = 0.25) | | | | R^2^ = 0.37/R^2^_adj_=0.34*** (f^2^ = 0.59) | | | |
| *Intercept* | *3415.461* | *598.445* | *5.707* | *<.001* | *3776.494* | *585.628* | *6.449* | *<0.001* |
| Age | -13.598 | 8.490 | -1.602 | 0.118 | **-19.132** | **8.308** | **-2.303** | **0.027** |
| Education | 155.619 | 102.348 | 1.520 | 0.137 | **252.665** | **100.156** | **2.523** | **0.016** |
| **Model 2** | R^2^ =0.47 /R^2^_adj_=0.42 (f^2^ = 0.89) | | | | R^2^ = 0.51/R^2^_adj_=0.47*** (f^2^ =1.04) | | | |
| *Intercept* | *3522.989* | *495.492* | *7.110* | *<0.001* | *3862.152* | *524.416* | *7.365* | *<0.001* |
| Age | -11.704 | 7.034 | -1.664 | 0.105 | **-17.623** | **7.445** | **-2.367** | **0.023** |
| Education | **175.811** | **84.763** | **2.074** | **0.045** | **268.751** | **89.711** | **2.996** | **0.005** |
| PI x Mapping | **-2.426** | **0.570** | **-4.256** | **<0.001** | **-1.932** | **0.603** | **-3.203** | **0.003** |
| **Model comparison** | | | | | | | | |
|  | **Left hippocampus** | | | | **Right hippocampus** | | | |
| **Models** | **∆R^2^** | **F** | **df1/df2** | **p** | **∆R^2^** | **F** | **df1/df2** | **p** |
| **1-2** | **0.27** | **18.114** | **1/36** | **<0.001** | **0.14** | **10.262** | **1/36** | **0.003** |

Abbreviations: Est.: Estimate; SE: Standard Error; PI: Path Integration. The variables with a significant (*p*<0.05) contribution to the model are marked in bold, and effect sizes are provided as Cohen’s f^2^. For the model fit measures, the number of stars designates the significance level: *** < 0.001, ** < 0.01, * < 0.05.

## Supplementary Information 6

We conducted a comprehensive set of diagnostic checks, including tests for normality of residuals, homoscedasticity, independence, multicollinearity, and influence diagnostics (Cook’s distance and Mahalanobis distance) for regression models for the hippocampus and entorhinal cortex. These diagnostics directly assess whether the assumptions underlying linear regression are met and whether individual observations exert disproportionate influence on the fitted models. Across all models, a small number of observations exceeded conservative χ²-based Mahalanobis distance thresholds. However, these cases were not associated with elevated Cook’s distance values and did not exert undue influence on parameter estimates.

**Model 1 (Left hippocampus ~ Age + Education + Visuospatial + Naming + Attention + Language + Abstraction + Delayed Recall + Orientation)**

For Model 1, residual normality was assessed using the Shapiro-Wilk test, which was not significant (*p* = 0.556), indicating no deviation from normality. Homoscedasticity was supported by the non-significant Breusch-Pagan test (*p* = 0.454). Independence of residuals was confirmed by a Durbin–Watson statistic (DW = 2.17, p = 0.632), indicating no autocorrelation. Multicollinearity was not evident, with Variance Inflation Factors (VIF) ranging from 1.09 to 2.31. Influence diagnostics indicated no observations that were unduly influential. Cook’s distance values ranged from 0.000 to 0.199 (mean = 0.032), and Mahalanobis distances ranged from 0.97 to 38.03, with only one observation exceeding a conservative χ² value of 27.88.

**Model 2 (Right hippocampus ~ Age + Education + Visuospatial + Naming + Attention + Language + Abstraction + Delayed Recall + Orientation)**

For Model 2, diagnostic checks indicated that regression assumptions were met. The residuals were normally distributed (Shapiro–Wilk, *p* = 0.459). Tests of homoscedasticity were non-significant (Breusch-Pagan: *p* = 0.447). The Durbin-Watson statistic indicated independent residuals (DW = 2.28, *p* = 0.394). Multicollinearity was low (VIF range = 1.09 – 2.31). No influential observations were detected (Cook’s distance range = 0.000 – 0.241, mean = 0.035; Mahalanobis distance range = 0.97 – 38.0, with one observation > χ² = 27.88).

**Model 3 (Left hippocampus ~ Age + Education + Maze Task + D-CAT + TMT-A + TMT-B + Dual-task)**

For Model 2, diagnostic checks indicated that regression assumptions were met. The residuals were normally distributed (Shapiro–Wilk, *p* = 0.538). Tests of homoscedasticity were non-significant (Breusch-Pagan: *p* = 0.965). The Durbin-Watson statistic indicated independent residuals (DW = 1.96, *p* = 0846). Multicollinearity was low (VIF range = 1.45 – 3.61). No influential observations were detected (Cook’s distance range = 0.000 – 0.260, mean = 0.037; Mahalanobis distance range = 1.03 – 29.35, with one observation > χ² = 24.32).

**Model 4 (Right hippocampus ~ Age + Education + Maze Task + D-CAT + TMT-A + TMT-B + Dual-task)**

For Model 2, diagnostic checks indicated that regression assumptions were met. The residuals were normally distributed (Shapiro–Wilk, *p* = 0.610). Tests of homoscedasticity were non-significant (Breusch-Pagan: *p* = 0.455). The Durbin-Watson statistic indicated independent residuals (DW = 2.32, *p* = 0.326). Multicollinearity was low (VIF range = 1.45 – 3.61). No influential observations were detected (Cook’s distance range = 0.000 – 0.225, mean = 0.036; Mahalanobis distance range = 1.03 – 29.35, with one observation > χ² = 24.32).

**Model 5 (Left hippocampus ~ Age + Education + VS Training + PI Distance + Pointing + Mapping + Perspective taking)**

For Model 5, diagnostic checks indicated that regression assumptions were met. The residuals were normally distributed (Shapiro–Wilk, *p* = 0.721). Tests of homoscedasticity were non-significant (Breusch-Pagan: *p* = 0.567). The Durbin-Watson statistic indicated independent residuals (DW = 2.14, *p* = 0.752). Multicollinearity was low (VIF range = 1.09 – 1.69). No influential observations were detected (Cook’s distance range = 0.000 – 0.329, mean = 0.039; Mahalanobis distance range = 0.67 – 26.58, with one observation > χ² = 24.32).

**Model 6 (Right hippocampus ~ Age + Education + VS Training + PI Distance + Pointing + Mapping + Perspective taking)**

For Model 6, diagnostic checks indicated that regression assumptions were met. The residuals were normally distributed (Shapiro–Wilk, *p* = 0.136). Tests of homoscedasticity were non-significant (Breusch-Pagan: *p* = 0.674). The Durbin-Watson statistics indicated independent residuals (DW = 2.21, *p* = 0.632). Multicollinearity was low (VIF range = 1.09 – 1.69). No influential observations were detected (Cook’s distance range = 0.000 – 0.958, mean = 0.051; Mahalanobis distance range = 0.67 – 26.58, with one observation > χ² = 24.32).

**Model 7 (Left hippocampus ~ Age + Education + PI*Pointing)**

For Model 7, diagnostic checks indicated that regression assumptions were met. The residuals were normally distributed (Shapiro–Wilk, *p* = 0.728). Tests of homoscedasticity were non-significant (Breusch-Pagan: *p* = 0.892). The Durbin-Watson statistics indicated independent residuals (DW = 2.10, *p* = 0.788). Multicollinearity was low (VIF range = 1.07 – 1.33). No influential observations were detected (Cook’s distance range = 0.000 – 0.204, mean = 0.032. Mahalanobis distance is not applicable since it can only be calculated with models that have two or more covariates.

**Model 8 (Right hippocampus ~ Age + Education + PI*Pointing)**

For Model 8, diagnostic checks indicated that regression assumptions were met. The residuals were normally distributed (Shapiro–Wilk, *p* = 0.411). Tests of homoscedasticity were non-significant (Breusch-Pagan: *p* = 0.361). The Durbin-Watson statistics indicated independent residuals (DW = 2.51, *p* = 0.090). Multicollinearity was low (VIF range = 1.07 – 1.33). No influential observations were detected (Cook’s distance range = 0.000 – 0.412, mean = 0.037. Mahalanobis distance is not applicable since it can only be calculated with models that have two or more covariates.

**Model 9 (Left hippocampus ~ Age + Education + PI*Map)**

For Model 9, diagnostic checks indicated that regression assumptions were met. The residuals were normally distributed (Shapiro–Wilk, *p* = 0.478). Tests of homoscedasticity were non-significant (Breusch-Pagan: *p* = 0.459). The Durbin-Watson statistic indicated independent residuals (DW = 2.21, *p* = 0.564). Multicollinearity was low (VIF range = 1.01 – 1.28). No influential observations were detected (Cook’s distance range = 0.000 – 0.130, mean = 0.029). Mahalanobis distance is not applicable since it can only be calculated with models that have two or more covariates.

**Model 10 (Right hippocampus ~ Age + Education + PI*Map)**

For Model 10, diagnostic checks indicated that regression assumptions were met. Residuals were normally distributed (Shapiro–Wilk *p* = 0.227). Tests of homoscedasticity were non-significant (Breusch-Pagan: *p* = 0.675). The Durbin-Watson statistic indicated independent residuals (DW = 2.08, *p* = 0.838). Multicollinearity was low (VIF range = 1.01 – 1.28). No influential observations were detected (Cook’s distance range = 0.000 – 0.100, mean = 0.026). Mahalanobis distance is not applicable since it can only be calculated with models that have two or more covariates.

**Model 11 (Left hippocampus ~ Age + Education + Naming + Orientation + PI*Map)**

For Model 11, diagnostic checks indicated that regression assumptions were met. The residuals were normally distributed (Shapiro–Wilk, *p* = 0.846). Tests of homoscedasticity were non-significant (Breusch-Pagan: *p* = 0.289). The Durbin-Watson statistic indicated independent residuals (DW = 2.28, *p* = 0.396). Multicollinearity was low (VIF range = 1.04 – 1.32). No influential observations were detected (Cook’s distance range = 0.000 – 0.632, mean = 0.042; Mahalanobis distance range = 0.11 – 38.03, with one observation > χ² = 20.52).

**Model 12 (Right hippocampus ~ Age + Education + Orientation + PI*Map)**

For Model 12, diagnostic checks indicated that regression assumptions were met. The residuals were normally distributed (Shapiro–Wilk, *p* = 0.242). Tests of homoscedasticity were non-significant (Breusch-Pagan: *p* = 0.816). The Durbin-Watson statistic indicated independent residuals (DW = 2.13, *p* = 0.714). Multicollinearity was low (VIF range = 1.28 – 1.32). No influential observations were detected (Cook’s distance range = 0.000 – 0.243, mean = 0.030; Mahalanobis distance range = 0.08 – 14.82, with two observations > χ² = 18.47).
